# Graphene (0002)/Diamond (111) Heterojunction with High Piezoresistive Response

**DOI:** 10.1002/advs.76096

**Published:** 2026-07-06

**Authors:** Xueyu Zhang, Kun Guo, Zhigang Gai, Tianxiao Guo, Yuan Gao, Jiancai Leng, Mei Zhang, Tonggang Jiu, Yibao Wang, Shousheng Liu, Xin Jiang

**Affiliations:** ^1^ State Key Laboratory of Physical Oceanography Institute of Oceanographic Instrumentation Qilu University of Technology (Shandong Academy of Sciences) Qingdao P. R. China; ^2^ Institute of Materials Engineering University of Siegen Siegen Germany; ^3^ International School For Optoelectronic Engineering Qilu University of Technology (Shandong Academy of Sciences) Jinan P. R. China; ^4^ Shandong Provincial Key Laboratory For Science of Material Creation and Energy Conversion Science Center for Material Creation and Energy Conversion Institute of Frontier and Interdisciplinary Shandong University Qingdao P. R. China

**Keywords:** diamond, graphene, heterostructures, piezoresistive

## Abstract

Diamond and graphene are emerging as promising successors to silicon‐based materials for semiconductor applications, particularly when integrated into graphene/diamond heterostructures. However, fabricating high‐quality graphene (0002) atomic layers on diamond (111) with intimate contact—mostly desired for device integration—remains a great challenge. Here, we report a strategy to realize a covalently bonded, graphene (0002)/diamond (111) heterojunction using thermal electron irradiation. Combining structural characterization with theoretical calculations, the formation mechanism and energy band characteristics of this junction structure were clarified, and the microscopic mechanism of hexagonal diamond (lonsdaleite) acting as an intermediate state before its transformation into graphene layers was proposed. The heterostructure exhibits a high piezoresistive response under the present testing configuration, with a gauge factor of −1149. This performance is attributed to two key factors: first, the heterojunction‐induced formation of an electron‐rich layer on the diamond surface; and second, a significant stress‐induced increase in the density of states of carbon C *2p* orbitals within the diamond layer. Leveraging diamond's intrinsic properties, piezoresistive chips based on this structure may offer opportunities for high‐temperature, radiation‐tolerant, wide‐range, and fast‐response sensing applications after further device‐level optimization and validation. These results provide a useful basis for developing diamond‐based semiconductor and sensing devices.

## Graphical abstract

Graphene/diamond heterostructures show promise as silicon alternatives for semiconductors, but high‐quality integration is challenging. Thermal electron irradiation enables covalent bonding; lonsdaleite acts as an intermediate. The heterostructure exhibits a high piezoresistive response (gauge factor −1149) associated with electron‐rich layers and stress‐induced DOS increase, showing potential for diamond‐based sensing chips.

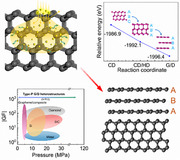

## 1 Introduction

Semiconductors underpin modern electronics [1, 2], yet silicon‐based materials are fast approaching intrinsic performance limits dictated by fundamental physics [3]. This looming constraint has driven an urgent search for alternatives—and synthetic diamond stands out as a leading candidate. Endowed with a wide bandgap, high thermal conductivity, and chemical inertness, it has spurred intense research targeting applications such as quantum computing, nanoscale sensing, and advanced electronics [4, 5, 6, 7]. Realizing its full semiconductor potential, however, requires overcoming key hurdles: stable, controllable n‐type doping remains a long‐standing bottleneck; heavy boron doping (for p‐type conductivity) induces severe lattice distortion that degrades crystallinity; and carrier recombination or scattering at grain boundaries/defects further limits device performance. While surface engineering advances—such as surface transfer doping and termination‐mediated conduction (e.g., with hydrogen or silicon)—have shown promise for mitigating conductivity limitations [8, 9, 10], tuning diamond's energy band structure via interface engineering remains a crucial, practical route to unlocking its applications.

Graphene, a distinct carbon allotrope, boasts complementary advantages: high electron mobility and tunable electronic properties [11, 12, 13]. These attributes have directed interest toward graphene/diamond (G/D) heterostructures as a strategy to overcome the intrinsic limitations of diamond while further enhancing the potential of graphene [14, 15, 16]. Combining graphene's zero‐bandgap Dirac cone structure with diamond's ultra‐wide bandgap yields an interface that enables charge redistribution, thereby tuning the system's Fermi level [17]. Additionally, this heterojunction interface is anticipated to overcome the limitation of graphene's “Dirac point electronic state deficit” —a property that further enhances graphene's application potential. This interface‐driven band structure engineering endows the heterostructure with potential for electronic devices—particularly those requiring integration with standard chip architectures, where heterostructures with graphene's 2D plane parallel to the diamond surface (type‐P) are ideal [13]. However, most current G/D junctions—synthesized via chemical vapor deposition (CVD) [16, 17], epitaxial growth [18], metal catalysis [19, 20], furnace annealing [21], or iron catalysis [22]—adopt a vertical (type‐V) configuration (graphene planes perpendicular to diamond). Despite theoretical predictions of tunable band gaps in type‐P G/D materials [23, 24, 25, 26, 27], high‐quality, strongly bonded type‐P heterostructures have remained elusive.

Structural compatibility between diamond and graphene suggests a pathway to type‐P growth: the (111) face of diamond and (0002) face of graphene exhibit near‐ideal lattice matching (C─C bond length discrepancy <2%; 1.45 Å vs. 1.42 Å) [28], implying the feasibility of direct transformation using diamond (111) as a template. As early as 1996, theoretical models proposed such a transformation [29], and high‐temperature/high‐pressure (HTHP) studies on graphite‐diamond phase transitions confirmed the structural link between graphite (0002) and diamond (111) faces, identifying micro‐heterostructures (type 1 and type 2) analogous to type‐P and type‐V [30, 31, 32, 33]. However, current approaches for fabricating type‐P G/D heterojunctions suffer from critical limitations: While transferring graphene onto diamond surfaces via the layer transfer method is seemingly straightforward, the resulting heterojunctions exhibit weak interfacial bonding and involve cumbersome processing; Vacuum annealing—also referred to as tube furnace‐induced processing in relevant literature—induces the graphitization of diamond, generating small‐sized and irregularly distributed few‐layer graphene (FLG) aligned with the (111) plane of diamond [14, 23, 28], a feature that restricts its practical utility. Notably, for type‐P G/D heterojunctions formed by vacuum annealing, the observation of the Dirac point at the Fermi level indicates weak graphene‐substrate interaction—consistent with the layer transfer method—along with an energy band structure analogous to that of single‐layer graphene [26]. Laser‐induced graphitization provides another nonequilibrium route for modifying diamond surfaces [34, 35]. In this process, the absorbed optical energy is rapidly converted into localized heat, which can promote sp^3^‐to‐sp^2^ carbon conversion in the irradiated region [36]. However, because the energy input is highly confined in space and time, laser treatment generally produces steep local temperature gradients and may be accompanied by localized graphitization, ablation, defect accumulation, or microstructural nonuniformity. Therefore, although laser‐induced graphitization is useful for local patterning or surface modification of diamond, it remains challenging to use this approach to construct large‐area, uniform, and strongly bonded graphene (0002)/diamond (111) heterojunctions. Weakly bonded heterostructures of this type inevitably encounter limitations in semiconductor applications. While covalently bonded G/D heterojunctions themselves are metastable, possess positive interfacial energy [17], and are extremely challenging to synthesize, their inherent properties remain highly appealing: beyond their potential for excellent mechanical performance, their electronic properties also exhibit distinct advantages. Notably, in the field of pressure sensors—where graphene is already widely used—the weak graphene‐substrate adhesion in weakly bonded heterojunctions directly constrains the performance ceilings and application scopes of devices; in contrast, covalently bonded type‐P G/D heterojunctions hold promise for fully unlocking the synergistic potential of graphene and diamond in terms of piezoresistive properties. Meanwhile, the microscopic mechanisms governing the transformation of type‐P G/D heterostructures remain incompletely elucidated. Specifically, for the tetrahedral carbon atomic configuration of cubic diamond, the exfoliation mechanism underlying graphene formation is insufficient to account for the generation of diverse microstructures—particularly in the absence of artificially applied gradient temperature fields. This factor further represents a critical constraint on the structural dimensions of heterojunctions.

Here we report on the fabrication of G/D heterostructures using a hot electron irradiation device with adjustable power. Systematic microstructural characterization confirms successful synthesis of high‐quality, type‐P G/D heterostructures that fully cover the diamond (111) face, with intimate, covalently bonded interfaces. Theoretical calculations clarify the transformation mechanism and predict feasibility for semiconductor applications. In particular, piezoresistive measurements reveal a high negative gauge factor (GF) for the heterojunction under the present testing configuration, indicating its potential for diamond‐based electromechanical sensing applications. This work demonstrates a strongly bonded type‐P G/D heterostructure and highlights its potential as a platform for diamond‐based semiconductor and sensing devices.

## 2 Results and Discussion

### 2.1 Synthesis and Microstructural Characterization of the G/D Heterojunctions

**FIGURE 1:**
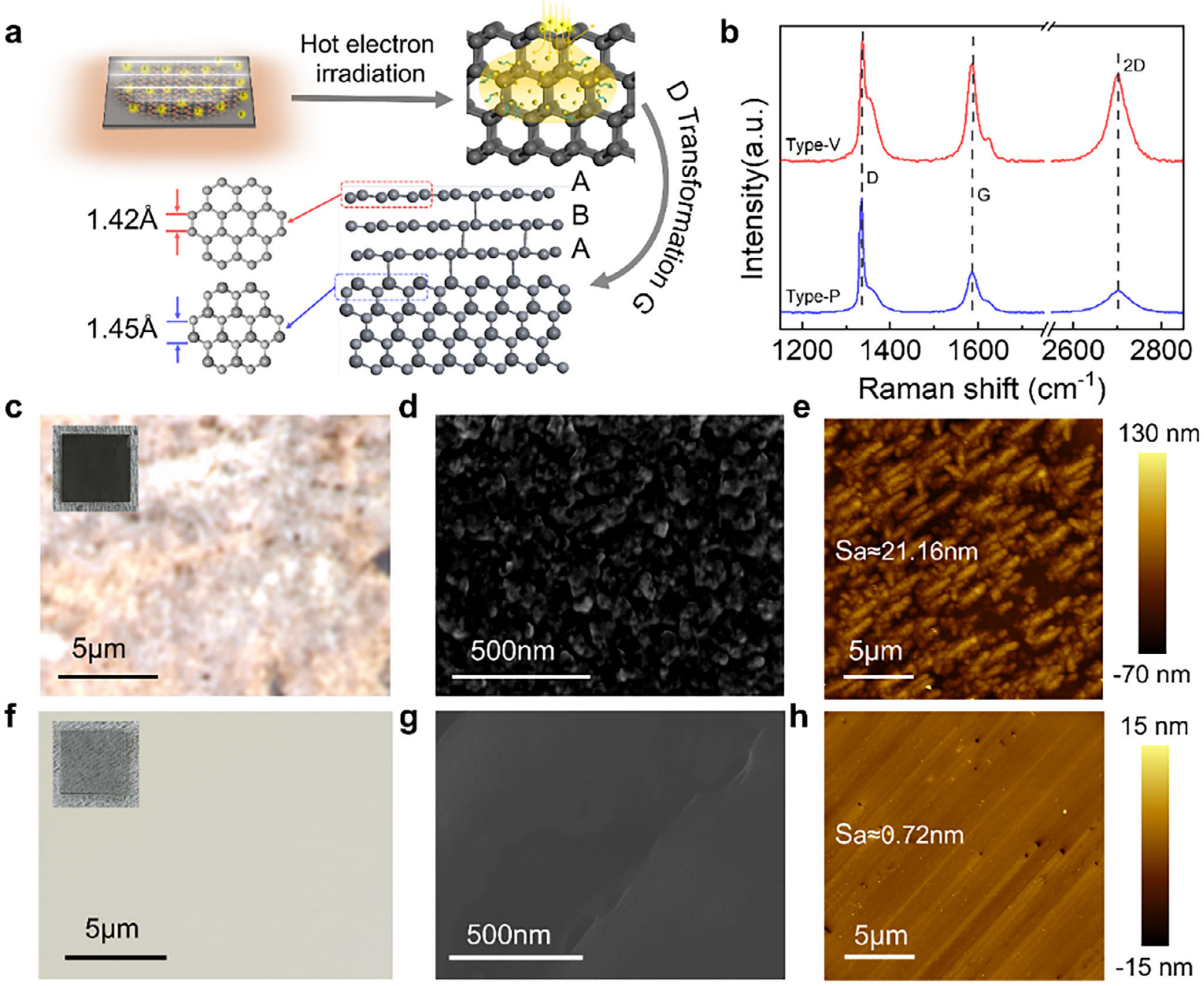
Preparation and surface structure characterization of G/D heterojunction. (a) Schematic illustration of the fabrication of type‐P G/D heterostructures via hot‐electron irradiation. (b) Raman spectra of different G/D heterostructures. (c–e) Optical microscopy, SEM, and AFM images of the type‐V structure, respectively. (f–h) Optical microscopy, SEM, and AFM images of the type‐P structure, respectively. The insets of Figure (c) and (f) are the physical photos of the heterostructure on dust‐free paper.

Figure 1a schematically illustrates the G/D heterojunction fabricated using the hot electron irradiation device (specific equipment and preparation parameters for type‐V and type‐P structures are detailed in the section Methods). Unlike conventional focused electron‐beam irradiation, such as keV‐level electron beams in TEM, the present method employs low‐energy hot electrons emitted from heated filaments and accelerated toward the diamond substrate by a positive bias under a low‐pressure hydrogen atmosphere [37]. This configuration provides a large‐area and relatively uniform near‐surface energy input coupled with a controlled thermal field, which is critical for activating the nanoscale subsurface layer while suppressing direct surface bond cleavage and the formation of vertically oriented graphitic structures. Figure 1b shows the Raman spectra of the G/D heterostructures with distinct configurations. Both exhibit characteristic peaks: the diamond sp^3^ peak at ∼1332 cm^−1^, and the graphene G (1580 cm^−1^) and 2D (2700 cm^−1^) peaks [38]. For reference, the bare single‐crystal diamond substrate displays only the 1332 cm^−1^ Raman peak (Figure S1). We characterized the surface morphology of type‐V (Figure 1c–e) and type‐P (Figure 1f–h) G/D heterostructures using optical microscopy, scanning electron microscopy (SEM), and atomic force microscopy (AFM). The type‐P heterostructure has a significantly smoother surface than type‐V. Under optical microscopy, the type‐P structure (Figure 1f) shows only slight transparency reduction relative to intrinsic single‐crystal diamond (Figure S1a). In contrast, the type‐V structure exhibits substantial topographical undulations in its surface graphene: SEM imaging (Figure 1d) reveals numerous flake‐like structures perpendicular to the substrate, whereas the type‐P structure (Figure 1g) features a flat, 2D graphene surface. AFM further differentiates the two: type‐V has a surface roughness (Sa) of 21.16 nm, while type‐P exhibits Sa = 0.72 nm—only marginally higher than that of intrinsic single‐crystal diamond (Sa = 0.65 nm; Figure S1c).

The type‐V structure is characterized by a thicker surface graphene layer, lower transparency, and higher roughness, consistent with the weakened intensity of the 1332 cm^−1^ peak (assigned to the diamond substrate) in its Raman spectrum. Analysis of the graphene G (1580 cm^−1^) and 2D (2700 cm^−1^) peaks indicates fewer layers in type‐V, with an I_2D_/I_G_ ratio of ∼1, corresponding to 1–2 layers. This appears contradictory to the results of the surface morphology characterization, but arises because of the parallel alignment of the graphene sheets with respect to the direction of the incident laser, with layer number decreasing upward. Thus, the thicker type‐V graphene layer results in a weaker diamond peak and a larger I_2D_/I_G_ ratio. In contrast, the type‐P structure has an I_2D_/I_G_ ratio of ∼0.7 (4–6 layers) while retaining a more prominent diamond characteristic peak.

**FIGURE 2:**
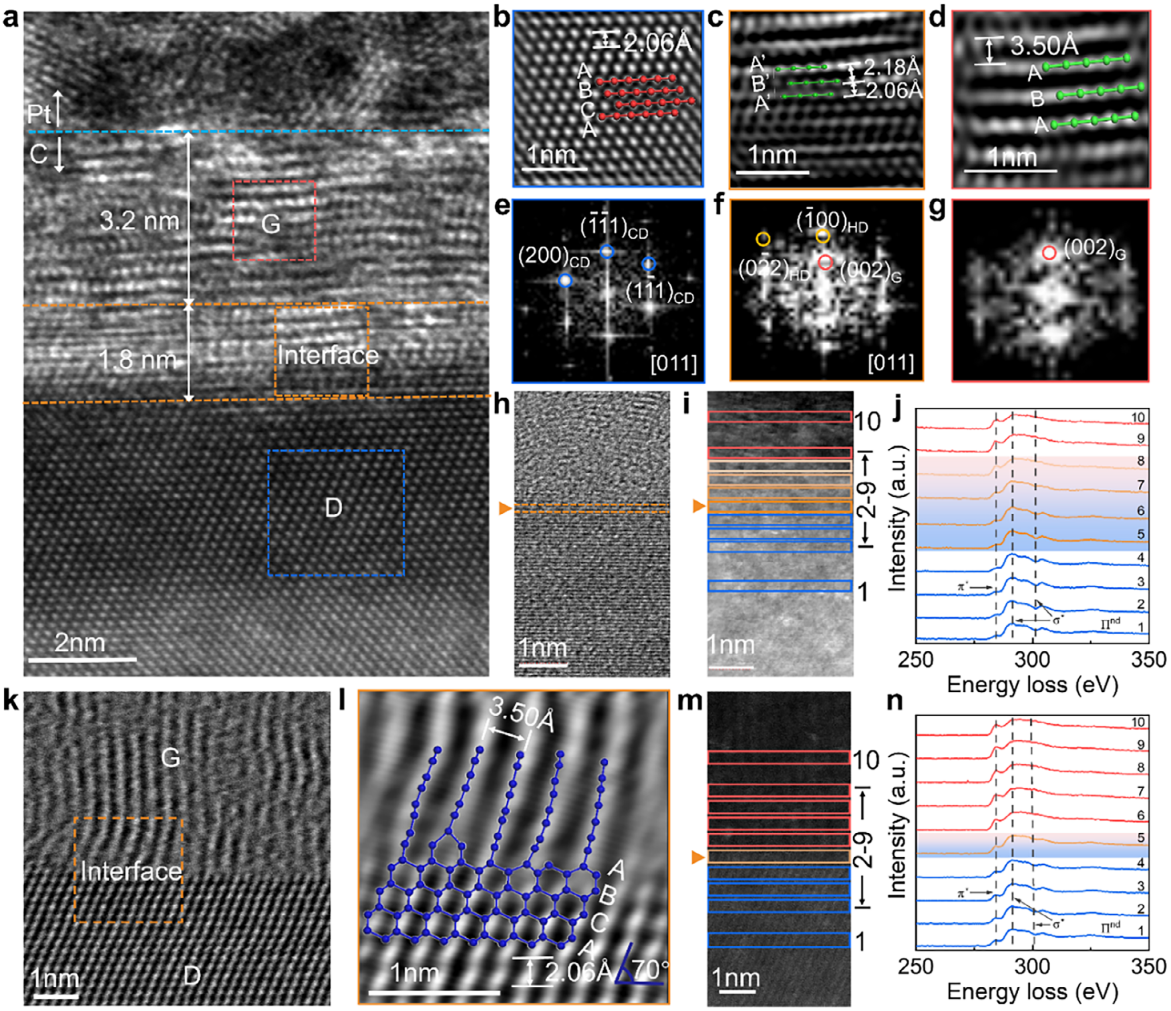
Microstructural characterization of different G/D heterojunctions by TEM. (a) Cross‐sectional TEM image of the type‐P structure. (b–d) High‐resolution bright‐field images of the diamond (blue), interface (yellow), and graphene (red) regions in the panel, respectively. (e–g) FFT patterns corresponding to panels (b–d), respectively. (h,i) Bright‐field and dark‐field TEM images of the regions characterized by EELS. (j) Core‐loss spectra acquired from the corresponding regions in panels (h) and (i). (k) Cross‐sectional TEM image of the type‐V structure. (l) High‐resolution image of the green region in panel (k). (m) Dark‐field TEM image of the type‐V structure from the EELS characterization. (n) Core‐loss spectra acquired from the corresponding regions in panel (m).

The structures and bonding states of the G/D heterostructures were characterized by transmission electron microscopy (TEM) combined with electron energy‐loss spectroscopy (EELS). Cross‐sectional specimens were prepared via focused ion beam (FIB) milling (Figures S2 and S3 for type‐V and type‐P, respectively). For the type‐P structure, a bright‐field (BF) TEM image of its cross‐section (Figure 2a) shows three distinct regions—single‐crystal diamond, interface, and graphene—demarcated by blue dashed lines. High‐resolution TEM (HRTEM) images (Figure 2b–d) reveal: a cubic diamond (CD) region with a ($\bar{1}\bar{1}$1) interplanar spacing of 2.06 Å and regular ABC stacking (Figure 2b); an interfacial region with expanded interlayer spacing (2.18 Å) matching hexagonal diamond (HD) ($\bar{1}$00) planes and A'B’A’ stacking (Figure 2c); and graphene layers with ABA stacking and 3.5 Å interplanar spacing (Figure 2d). Corresponding fast Fourier transform (FFT) patterns (Figure 2e–g) confirm these structures, with Figure 2e aligning with diamond's [011] zone axis diffraction.

To gain deeper insights into the bonding states of carbon atoms within the composite structure, particularly at the interface, we performed high‐resolution EELS analysis at the covalently bonded interface of the type‐P composite using aberration‐corrected TEM. Figure 2h shows a BF‐TEM image, and Figure 2i presents an atom‐resolved high‐angle annular dark‐field (HAADF) scanning TEM (STEM) image, where regions 1–4 correspond to diamond, 5–7 to the interface, and 8–10 to graphene (from bottom to top). Figure 2j displays core‐loss EELS spectra acquired from the respective regions in Figure 2i: In regions 1–4, the spectra are dominated by a distinct σ* peak at 292 eV and a prominent dip at 302 eV, characteristic of sp^3^‐hybridized carbon in diamond [39, 40]. In contrast, the π* peaks associated with sp^2^‐hybridized carbons at 285 eV are barely discernible. In region 5, corresponding to the uppermost layer of the cubic diamond substrate at the interface, the 302 eV dip (characteristic of cubic diamond) and σ* peak remain clearly visible. Notably, this spectral feature persists in region 6, which is even further from the diamond surface. In region 7, the 302 eV dip is absent, though the σ* peak remains distinct, with no significant elevation of the π* peak. Moving to regions 8–10, the spectra exhibit a prominent π* peak at 285 eV and a progressively weaker σ* peak at 292 eV, typical of graphitic *sp^2^*‐hybridized carbon.

For comparison, type‐V HRTEM images (Figure 2k–l) show an abrupt graphite‐diamond interface with 5:8 commensurability between graphene (0002) and diamond {111} planes (similar to reports in the literature [17, 19]). EELS analysis of the type‐V structure (Figure 2m) reveals similar partitioning into 10 regions: Regions 1–4 (diamond) exhibit σ* peaks at 292 eV and 302 eV dips, with negligible π* peaks, analogous to the type‐P structure. However, in region 5 of the type‐V structure, the 302 eV dip is significantly attenuated. More strikingly, the EELS spectrum in region 6 closely resembles that of the overlying graphene, indicating that the phase transition in type‐V occurs over an extremely narrow range (∼1–2 atomic layers). Overall, as the carbon bonding transitions from sp^3^ (diamond) to sp^2^ (graphene), the π* peak intensity in EELS spectra increases while the σ* peak diminishes. This transition is confined to the interfacial region (region 5), where the EELS spectrum retains diamond‐like features, indicating that the covalently bonded type‐V structure has an interfacial region with mixed sp^3^/sp^2^ carbon bonding.

### 2.2 Formation Mechanism of Type‐P G/D Heterojunctions

**FIGURE 3:**
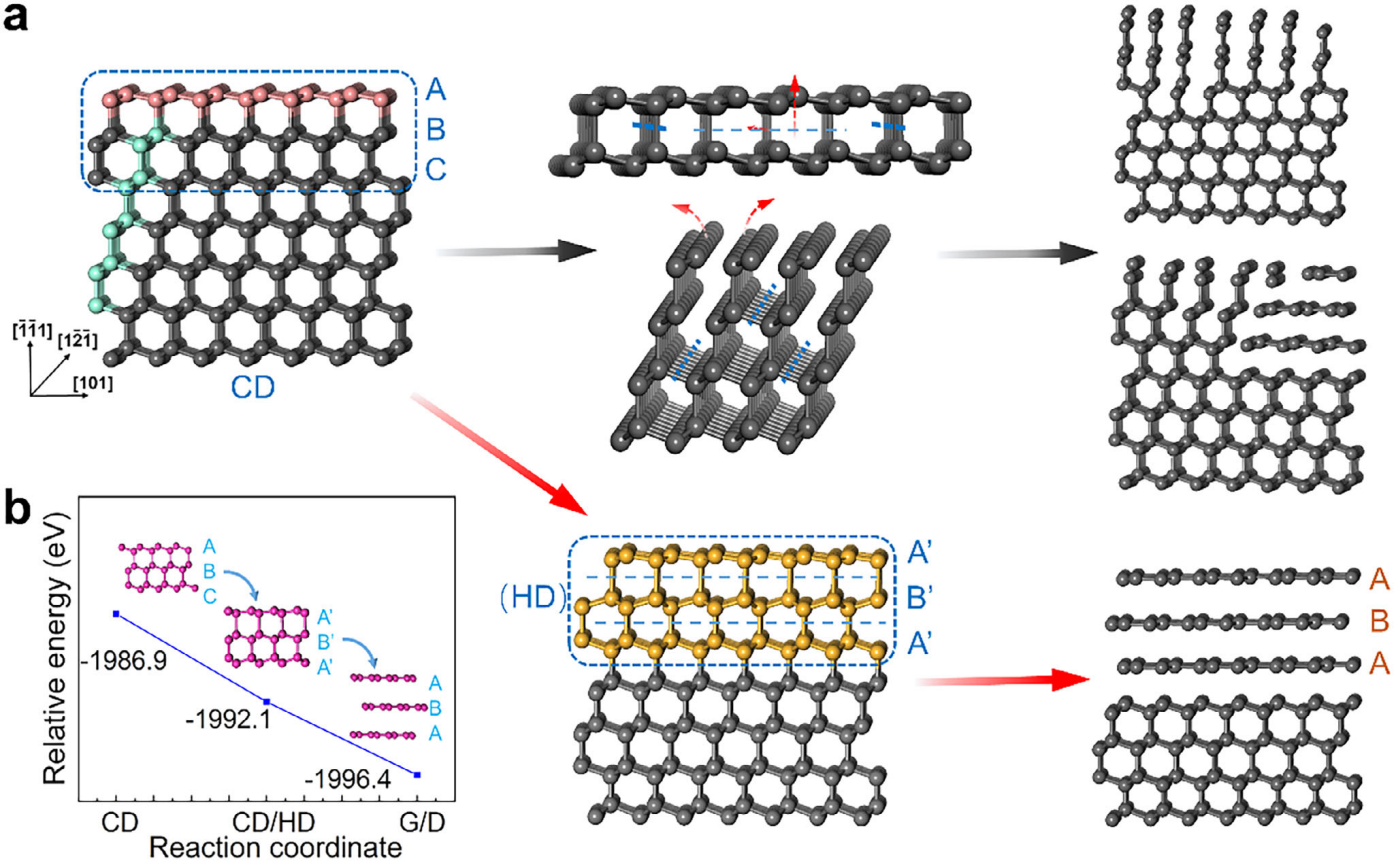
Formation mechanism of G/D Heterojunction. (a) Schematic diagram of G/D heterojunction formation via surface phase transition of CD. (b) Calculated relative atomic energy variations of carbon atoms in different structures.

In comparison to type‐V heterojunctions, the formation of type‐P G/D heterostructures on diamond surfaces has rarely been reported. The intrinsic transformation relationship between diamond (111) faces and graphite (0002) faces has been widely acknowledged in the scientific community [14, 19, 29, 30, 31], and this inherent lattice matching mechanism constitutes the key rationale for selecting single‐crystal diamonds with {111} face families as substrates in G/D heterojunction fabrication. Theoretical studies on the electronic band properties of type‐P G/D interfaces have revealed that graphene can form on diamond (111) surfaces through high‐temperature exfoliation, a formation pathway corroborated by relevant research [23, 26]. Beyond the aforementioned lattice matching characteristics, the {111} face family of CD exhibits the largest interplanar spacing and the lowest areal density of covalent bonds between faces, serving as the cleavage plane of CD. This further substantiates the layer‐exfoliation transformation mechanism of (111) faces. However, as illustrated in Figure 3, single‐crystal diamonds with 3D structures contain {111} face families with multiple orientations. For instance, the (1$\bar{1}$1) face, which forms an approximate 69° angle with the surface ($\bar{1}\bar{1}$1) face, also possesses a high degree of lattice matching with the graphite (0002) face. This inevitably leads to competitive growth between different types of heterojunctions during the graphitization phase transition of diamond under vacuum heating: specifically, type‐V structures extend inward after surface chemical bond cleavage, exhibiting 1D growth characteristics; in contrast, type‐P structures require achieving chemical bond cleavage beneath the surface atomic layer while avoiding bond breaking in the outermost layer, with development along the 2D planar direction. In comparison, type‐V structures initiate from the outermost surface bond cleavage, with multiple {111} facets extending unidirectionally and simultaneously inward, whereas type‐P structures involve 2D expansion of a single facet to the periphery. Evidently, vertically growing type‐V structures form more readily. On the other hand, when the dimensions of diamond are reduced to the nanoscale, the aforementioned competitive growth phenomenon vanishes, and graphene structures exfoliate along a single {111} face family to form microscale type‐P structures, which become the dominant morphology. This explains why type‐V structures constitute the primary form of G/D heterojunctions on large‐sized diamond substrates, while observed type‐P structures are relatively small in size (typically below the micrometer scale) [14].

In fact, during heterojunction fabrication, if interfacial activity is further enhanced—for example by hydrogen plasma etching, plasma‐enhanced chemical vapor deposition (PECVD) or other methods—to introduce or induce the evaporation of surface carbon atoms on diamond, forming active carbon species that subsequently reconstitute into graphite (graphene), the kinetic advantage of type‐V structures becomes even more pronounced (Figure S4) [16, 17, 21]. Consequently, technologies for producing large‐scale (millimeter‐sized) type‐P G/D heterojunctions have yet to achieve a breakthrough. To our knowledge, there are currently no reports of covalently bonded type‐P G/D heterojunctions.

The transformation induced by hot‐electron irradiation can also be regarded as a nonequilibrium process, similar in a broad sense to laser‐induced graphitization of diamond [34, 35]. Both processes involve external energy input, defect‐assisted bond rearrangement, and partial conversion from sp^3^‐to‐sp^2^‐bonded carbon. However, their energy‐deposition characteristics are different. Laser treatment is usually dominated by highly localized and transient thermal effects, where steep temperature gradients can promote surface graphitization, local ablation, and defect accumulation [36]. By contrast, hot‐electron irradiation provides a more spatially extended electron flux under a controlled thermal field. This feature allows the near‐surface and nanoscale subsurface region of diamond to be activated without inducing extensive surface bond cleavage. In the hot electron irradiation method used in this work, electrons penetrate the nanoscale subsurface layer and undergo elastic or inelastic collisions with the underlying carbon atoms. Notably, the temperature field does not decrease gradually from the diamond surface toward the interior; instead, a 2D active layer forms beneath the surface layer. By controlling the surface temperature below the threshold for C‐C covalent bond cleavage, the formation of the type‐V structure is suppressed, thereby enabling the fabrication of large‐area, uniform type‐P heterojunctions. The type‐P processing window is narrow: exceeding the critical electron energy cleaves surface bonds, which then propagate inward to form type‐V heterojunctions (Figure S5). Additionally, high‐quality diamond substrates are essential as grain boundaries and defects serve as preferential graphitization sites, disrupting uniformity (Figure S6).

Prior to the successful fabrication of the type‐P structure, it is necessary to address the transition between the ABC stacking of the CD {111} plane family and the AB stacking of few‐layer graphene. This issue also persists in the preparation of graphite and diamond materials, representing a long‐standing common challenge. Studies on the graphite‐to‐diamond phase transition in bulk materials propose two pathways involving layered structural transitions: (a) the slipping of ABA‐stacked graphite layers to form ABC stacking, followed by conversion to CD under high temperature and pressure; and (b) the transformation of ABA‐stacked graphite to HD, which subsequently converts to CD [41]. In both cases, ABC‐stacked graphite and HD serve as key intermediates that facilitate the stacking transition from ABA to ABC. In our experiments, the A'B’A’‐stacked carbon layers at the type‐P G/D interface (Figure 2c) exhibit an interlayer spacing smaller than graphite's characteristic 3.5 Å, matching the (100) plane spacing of HD. Based on these observations, we propose a roadmap for the transformation of CD to ABA‐stacked graphene and have constructed three structural models—CD, CD/HD composite, and graphene/CD (G/D) (Figure S7)—to analyze their relative atomic energies. The theoretical equilibrium structure of the CD/HD composite is shown in Figure S6. As shown in Figure 3b, the relative atomic energies decrease sequentially from CD to CD/HD composite to G/D, indicating that the HD intermediate likely reduces the phase transition barrier and improves transformation efficiency, thereby driving the system from the higher‐energy CD state toward the more stable, lower‐energy graphene configuration. By integrating experimental and theoretical findings, we propose the formation mechanism of the type‐P structure: under hot electron irradiation, subsurface CD atoms first transition from the ABC‐stacked cubic structure to A'B'A'‐stacked HD. With prolonged electron bombardment, the σ bonds perpendicular to the (100) plane of HD break, inducing partial sp^3^‐to‐sp^2^ hybridization and ultimately leading to the formation of the upper ABA‐stacked graphene layers (Figure 2d). Notably, HD—typically a minor phase formed under extreme conditions, with its synthetic preparation only recently reported [39, 41]—emerges herein as a critical intermediate, extending its previously recognized role in graphite‐diamond transitions.

### 2.3 Piezoresistive Properties of Type‐P G/D Heterojunctions

**FIGURE 4:**
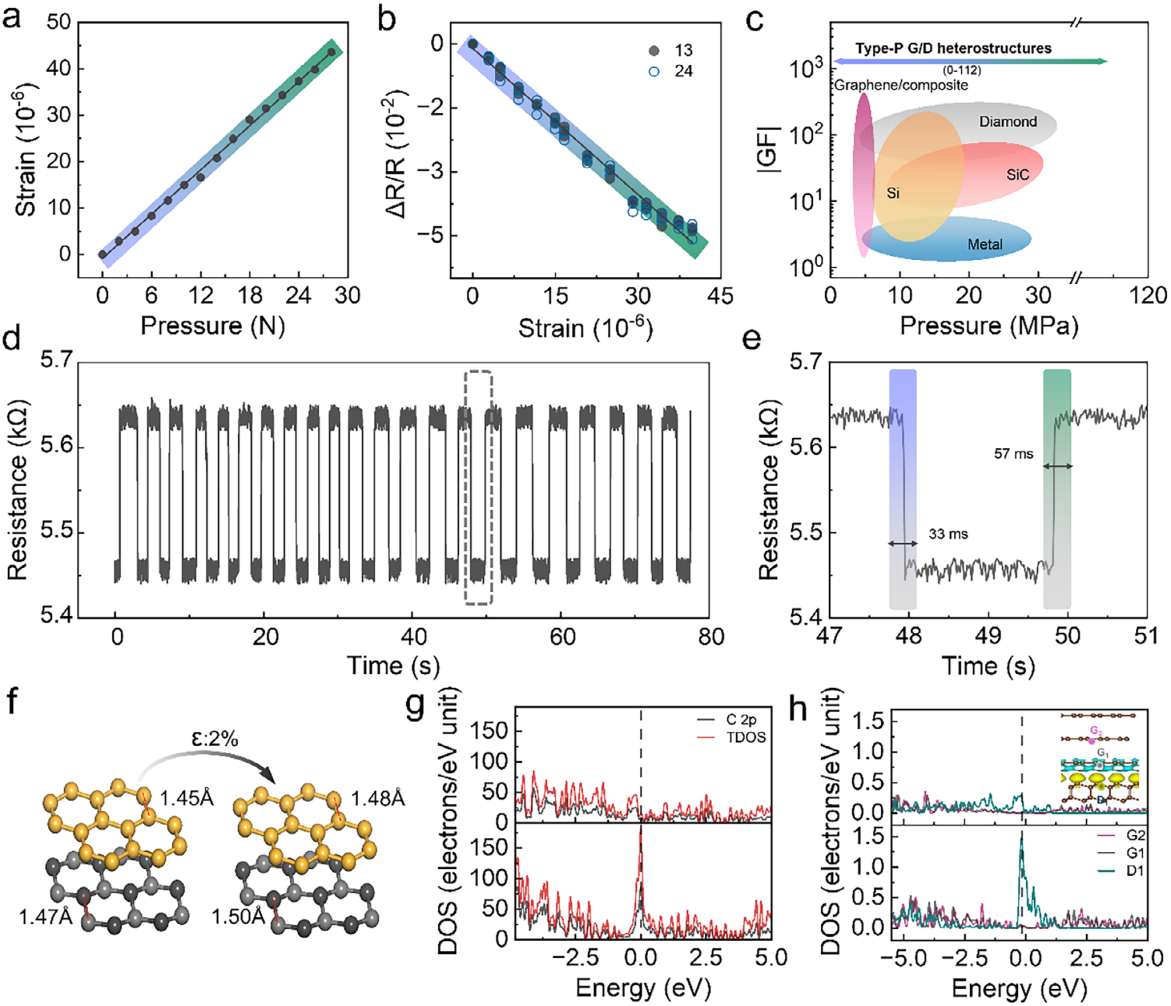
Piezoresistive performance characterization. (a) The relationship between strain (ε) and pressure of the heterostructure obtained from finite element analysis calculations. (b) Relative resistance change (ΔR/R_0_) of the type‐P G/D heterostructures as a function of the strain, along with the calculated GF. (c) GF and strain for type‐P G/D and other piezoresistive materials in the literature. (d) and (e) Resistance variation and response time of the type‐P G/D structure under cyclic loading with 28 N pressure. (f) Diagram of crystal structure changes of type‐P G/D heterojunction with and without 2% biaxial tensile strain. (g) DOS variation of the type‐P G/D heterostructures under 2% strain. (h) LDOS for specific carbon atoms in the graphite and diamond phases with and without 2% biaxial tensile strain. Inset: Charge density difference at the type‐P G/D interface; green and yellow isosurfaces represent charge depletion and accumulation in space, respectively.

To explore the potential of the as‐prepared type‐P G/D heterojunction in semiconductor applications, we systematically investigated its piezoresistive properties. Piezoresistivity not only reports on changes in the heterojunction's electrical performance under strain—a core characteristic of semiconductors for such applications—but also enables validation, via cyclic stress‐loading experiments, of a key advantage of our fabrication strategy over conventional approaches (e.g., layer transfer, exfoliation). Specifically, this advantage stems from the superior structural stability afforded by strong covalent interfacial bonding. Figure S9 shows a photograph of the custom‐built probe station used for piezoresistive performance testing, as well as a schematic diagram of the piezoresistive performance measurement of the heterojunction electrode. Herein, electrodes 1–3 and 2–4, aligned along diagonal axes, were selected for analysis. The GF, a critical metric for piezoresistive performance, is defined as:

(1) $$\mathrm{GF}=\frac{\Delta R/R_{0}}{\varepsilon }$$

where *ΔR/R* is the relative resistance change and *ɛ* the applied strain. A higher GF confers greater signal modulation under smaller deformation, a prerequisite for high‐sensitivity, high‐resolution device design and construction. For the G/D heterojunction, the strain *ɛ* was derived from stress distributions via finite element simulations, as shown in Figure 4a. Given that the heterostructure is dominated by a 0.3‐mm‐thick single‐crystal diamond layer and the pressure is applied to the diamond side, Young's modulus E was set to that of single‐crystal diamond (1050 GPa). The pressure range was recalculated according to the actual loading parameters. With a maximum applied force of 28 N and an effective indenter contact area of 0.25 mm^2^, the maximum applied pressure is 112 MPa. Thus, the working pressure range of the present device is corrected to 0–112 MPa. Figure 4b shows the relationship between relative resistance variation (along distinct diagonal directions) and strain. Using Equation (1), the GF of the type‐P G/D heterostructure was calculated as −1149. This result shows a large negative piezoresistive response under the present testing configuration.

Figure 4c compares the GF and measurement ranges of the heterojunction with those of other piezoresistive materials reported in the literature [42, 43, 44, 45, 46, 47, 48, 49, 50, 51, 52, 53, 54]. When comparing GF values across different piezoresistive systems, the allowable strain window, strain‐to‐failure limit, working mechanism, and target application scenario should be considered. The type‐P G/D heterojunction in this work operates in a small elastic strain regime under the present loading conditions, which is far below the failure strain of single‐crystal diamond and is typical of rigid semiconductor pressure sensors. In contrast, flexible graphene‐based composite sensors can exhibit extremely high apparent GF values, reaching 10^4^–10^6^ in some cases [55, 56], but these values are usually obtained under large tensile or bending strains and are strongly associated with contact‐resistance variation, crack opening/closure, tunneling‐path evolution, or conductive‐network reconstruction. Therefore, direct numerical comparison of GF values without distinguishing strain regime and sensing mechanism may be incomplete. In this context, the GF of −1149 observed for the type‐P G/D heterojunction is more appropriately compared with rigid semiconductor piezoresistive materials, while its wide pressure range and high stiffness further support its potential for diamond‐based pressure‐sensing applications.

Mainstream pressure‐sensing materials (e.g., metals, silicon‐based materials, graphene, and their composites) face a “sensitivity‐measurement range‐stability” trade‐off: high‐sensitivity sensors typically suffer from narrow measurement ranges and poor environmental stability, while wide‐range sensors struggle to achieve high precision. It should be noted that GF values reported for different piezoresistive systems are not always directly equivalent because the measured values depend strongly on the loading mode, device structure, electrode configuration, and strain‐calculation method [42]. For example, in flexible graphene films and graphene‐based composites, the resistance variation may be greatly enhanced by structural changes such as wrinkle flattening, crack propagation, flake‐to‐flake contact variation, and conductive‐network reconstruction [52, 53, 54]. These effects can lead to very high apparent GFs, sometimes reaching values of several thousand or higher, but they are often accompanied by a limited strain or pressure range and a strong dependence on device architecture. In contrast, the present G/D heterojunction is fabricated on a bulk single‐crystal diamond chip, and the strain used for GF calculation is obtained from finite element analysis based on the actual loading geometry. Therefore, the GF of −1149 reported here reflects the electromechanical response of a rigid, covalently bonded semiconductor‐like heterostructure under a well‐defined loading condition, making the comparison with conventional piezoresistive materials more transparent.

Metal strain gauges, which rely on geometric deformation to induce resistance changes, exhibit a relatively low GF of ∼2, making them unsuitable for high‐precision and wide‐range applications [42]. By contrast, semiconductors exploit resistivity variation effects: external forces induce lattice distortion, altering energy band characteristics [43], which directly modifies resistivity and thus resistance. As representative semiconductors, silicon‐based materials leverage this piezoresistive property—for example, single‐crystal silicon exhibits a GF ranging from single‐digit values to 200 depending on its crystal orientation and doping conditions [42]. However, their inherent limitations restrict performance in demanding scenarios such as high‐temperature environments, wide measurement ranges, and high‐sensitivity applications. Silicon carbide (SiC), boasting superior mechanical properties (Young's modulus: 424 GPa; sublimation temperature: 1800°C), high thermal conductivity, and corrosion resistance, is a promising candidate for MEMS and NEMS devices. However, its GF (∼30) is comparable to that of silicon‐based materials, limiting its performance edge [44, 45]. Though optoelectronic coupling has enhanced SiC's GF from 20 to 58 000 [44], this improvement depends on external conditions (not intrinsic material properties), and device fabrication using this effect remains technically challenging. Boron‐doped diamond (BDD) films prepared via chemical vapor deposition (CVD) are an exception, with reported GFs exceeding 4000 [46]. Notably, however, polycrystalline BDD exhibits a GF of ∼283 (consistent with our previous work [47]), whereas individual grains (intra‐grain) reach 4000—comparable to single‐crystal diamond. Despite the recognized piezoresistive potential of doped single‐crystal diamond, millimeter‐scale doped single‐crystal diamond semiconductor devices remain unfabricated. Additionally, although graphene‐based materials and their composites offer high sensitivity, their intrinsic flexibility restricts them to narrow measurement ranges (<60 kPa) [53, 54].

Other piezoresistive materials (Table S1) show similarly constrained GFs: zinc oxide (ZnO) nanowires achieve a GF of 1250 via the piezoelectric effect, and most other piezoresistive materials/structures exhibit GFs ≤ 900. It can be seen that the type‐P G/D heterojunction shows considerable advantages in both GF and measurement range compared with major piezoresistive materials. Additionally, the heterojunction device exhibits good short‐term cyclic stability and reproducibility in the present cyclic tests (Figure 4d), with response times of 33 ms (pressurization) and 56 ms (depressurization), respectively (Figure 4e). Given the intrinsic bulk characteristics of diamond itself and the covalent bonding characteristics between the G/D heterojunction, this performance is consistent with the expectations based on its intrinsic properties. From the perspective of device applications, the uniformity and reproducibility of the G/D heterojunction are also critical. The present device was fabricated on a 3 × 3 × 0.3 mm single‐crystal diamond chip, rather than on a randomly assembled micro/nanoscale structure. The hot‐electron irradiation process provides a spatially extended energy input over the sample surface, which is favorable for uniform conversion across the millimeter‐scale diamond chip. This is supported by the surface characterization results: the type‐P G/D heterojunction exhibits a smooth and continuous surface, with an AFM roughness of 0.72 nm, which is close to that of pristine single‐crystal diamond. The use of high‐quality single‐crystal diamond further minimizes preferential graphitization at grain boundaries or extended defects, while the covalently bonded interface reduces the possibility of interfacial sliding or delamination during repeated loading. These factors contribute to the stable and reproducible cyclic piezoresistive response. Although wafer‐scale processing and systematic device‐to‐device statistics remain to be further investigated, the non‐focused and large‐area nature of hot‐electron irradiation suggests promising scalability for diamond‐based device fabrication.

### 2.4 Mechanisms Underlying the Piezoresistive Properties

To investigate the effect of strain on the piezoresistive properties of the heterojunction, we constructed a theoretical model of the G/D hybrid structure under 2% biaxial tensile strain (Figure S10), building on our previous theoretical framework and incorporating practical observations from piezoresistive property tests of the heterojunction. Figure 4f shows the change in carbon‐carbon (C─C) bond lengths of the two lattice layers at the graphene/diamond interface under 2% biaxial tensile strain; the C─C bond lengths in both the graphene and diamond phases exhibit a certain degree of elongation. Concurrently, a pronounced shift in the density of states (DOS) near the Fermi level is observed under strain (Figure 4g), indicating a significant change in the material's electrical conductivity. The charge density difference map of the type‐P structure (Figure 4h) shows electron depletion (green isosurfaces) on the graphene side and accumulation (yellow isosurfaces) at the diamond surface across the interface. This redistribution arises from interfacial interactions, with electron transfer from graphene to the diamond subsurface directly underpinning the composite's semiconducting behavior. To clarify the mechanism of strain‐induced DOS changes, we examined the local density of states (LDOS) for carbon atoms at distinct positions (Figure 4h). The LDOS distributions of the G_1_ and G_2_ carbon atoms from the graphene layer are similar, consistent with graphene's characteristic lack of electronic states at the Fermi level. In contrast, the D_1_ carbon atoms from the diamond layer exhibit a distinct LDOS distribution at the Fermi level. Under unstrained conditions, the LDOS distribution of D_1_ lies slightly below the Fermi level; under biaxial strain, the LDOS of D_1_ increases substantially (by approximately a factor of three) and shifts entirely toward higher energy levels. This characteristic provides a theoretical basis for the high piezoresistive response observed in the present testing configuration.

To further clarify the connection between the experimental interfacial characterization, theoretical predictions, and measured piezoresistive response, we note that the atomically resolved TEM images confirm the coherent graphene (0002)/diamond (111) interfacial configuration, while the EELS results reveal the coexistence and gradual transition of sp^2^‐ and sp^3^‐bonded carbon across the interface, providing experimental evidence for strong covalent interfacial coupling. This is consistent with the DFT‐calculated charge‐density difference, which shows interfacial charge redistribution. The covalently bonded interface also enables efficient strain transfer from the diamond substrate to the graphene/diamond interfacial region. Under tensile strain, DFT structural relaxation shows elongation of interfacial C─C bonds (Figure S10), accompanied by an increase in the DOS/LDOS near the Fermi level, especially for carbon atoms close to the diamond side of the interface. Therefore, the TEM–EELS evidence of covalent sp^2^/sp^3^ interfacial bonding, the DFT‐predicted charge redistribution and strain‐induced DOS/LDOS enhancement, and the macroscopically measured negative resistance change under loading form a consistent experimental–theoretical correlation, supporting that the piezoresistive response of the type‐P G/D heterojunction is closely associated with the hybrid carbon bonding network at the covalently bonded interface rather than originating solely from weak interfacial contact or classical geometric effects.

A critical distinction in piezoresistive behavior lies in the sign of the GF: metals and most semiconductors exhibit a positive piezoresistive factor. Specifically, under tensile strain (ε > 0), their resistance increases (ΔR/R_0_> 0); under compressive strain (ε < 0), resistance decreases (ΔR/R_0_< 0). This trend arises not only from strain‐induced modifications to the semiconductor band structure but also aligns with resistance changes driven by stress‐induced geometric variations—e.g., in composites of 2D materials like graphene, tensile strain increases interlayer spacing and reduces contact area, thereby elevating resistance [52, 53]. In contrast, the type‐P G/D heterojunction presented herein exhibits a large negative GF of −1149 under the present measurement configuration. While negative GFs have been documented for monocomponent materials (e.g., MoS_2_), monocomponent graphene and BDD both exhibit positive GFs: for these materials, strain application reduces Fermi velocity and carrier mobility, which in turn increases resistivity [49]. This observation highlights the uniqueness of the heterojunction's piezoresistive response—attributed to the intrinsic properties of the heterojunction structure, rather than the performance of its individual components. The high negative GF of the type‐P G/D heterojunction stems from two synergistic factors: First, the single‐crystal diamond substrate (Young's modulus: 1050 GPa) minimizes strain under external forces, ensuring that resistance variations are dominated by electronic effects rather than geometric deformation. This relatively small strain also prevents significant degradation of carrier mobility. Second, carrier redistribution occurs at the heterojunction interface, while deformation of the composite structure induces a substantial increase in the DOS. These two effects collectively play a crucial role in reducing resistance.

Beyond the observed high negative GF, the type‐P G/D heterojunction shows several promising features for further device exploration: (1) Measurement range: The operating pressure range of the heterojunction is corrected to 0–112 MPa based on the actual loading force and effective contact area, indicating its potential for wide‐range pressure sensing. (2) Measurement robustness: Tests were conducted on bulk samples (dimensions: 3 × 3 × 0.3 mm), avoiding the stochasticity inherent to micro/nano‐scale structures. Uniform pressure variation during measurements, combined with the large dynamic range, ensures excellent linearity, repeatability, and stability—validated via multiple replicate experiments. (3) Compatibility and physical model integration: Unlike many studies (Table S1) that report “material sensitivity” (due to challenges in deriving strain ε from Young's modulus for 2D flexible materials and their composites), the heterojunction is compatible with traditional semiconductor electrode architectures. It also rigorously satisfies the criteria for high GF as defined in Equation (1), enabling direct integration with semiconductor physical models. Combined with diamond's inherent properties—ultrahigh hardness, corrosion resistance, and environmental stability—the heterojunction can simultaneously address the “sensitivity‐measurement range‐stability” trilemma of piezoresistive materials, thereby holding enormous application potential in the fields of high‐precision and wide‐range pressure sensors, as well as in application scenarios under extreme environments (e.g., high temperatures, radiation fields).

To evaluate the influence of environmental temperature on the electrical response of the G/D heterojunction, the resistance‐temperature characteristics were measured from 25°C to 120°C (Figure S11). The temperature was increased in steps of 5°C, and the steady‐state resistance was recorded after the sample reached thermal equilibrium at each temperature. The results show that the resistance of the heterojunction decreases approximately linearly with increasing temperature, with a total resistance change of approximately 284 Ω. This temperature‐dependent response indicates that temperature drift should be considered in practical pressure‐sensing applications. The result also provides an experimental basis for developing temperature‐compensation strategies for future G/D‐heterojunction‐based sensing chips. In addition, humidity may influence the surface adsorption behavior and conductivity of exposed graphene‐based devices. For practical Wheatstone‐bridge pressure‐sensing chips, device packaging or sealing is expected to reduce the direct influence of environmental humidity on the graphene surface; nevertheless, systematic humidity‐dependent measurements of unsealed and packaged devices will be valuable in future work.

## 3 Conclusion

In summary, type‐P G/D heterojunctions were successfully fabricated on single‐crystalline diamond (111) faces using thermal electron irradiation. This fabrication method elegantly circumvents the inherent complexity and potential material damage associated with layer‐transfer processes while significantly enhancing the interfacial bonding strength of the graphene to diamond. Through systematic characterization of the microstructure and in‐depth analysis of theoretical models, we clearly unravel the phase transition pathway from cubic diamond to graphene. Owing to the significant changes in the density of states near the Fermi level under strain, this junction structure exhibits a high piezoresistive response under the present testing configuration. Combined with diamond's extraordinary physicochemical properties, this novel structure holds substantial potential for development in high‐precision, wide‐range pressure sensors—particularly for pressure detection applications in specialized extreme environments. Beyond piezoresistive applications, the covalently bonded G/D interface may also have potential implications for interfacial thermal transport and diamond‐based thermal management. Diamond is widely valued for its ultrahigh thermal conductivity, while graphene can provide efficient in‐plane heat spreading. Compared with weakly bonded or transfer‐formed graphene/diamond interfaces, the covalent bonding configuration obtained here may reduce interfacial voids, contamination layers, and weak‐contact‐induced phonon scattering, which are important factors governing interfacial thermal resistance and interface stability. Such a carbon‐based covalent interface may therefore provide an interesting platform for coupling lateral heat spreading with vertical heat dissipation, particularly in the context of integration with wide‐bandgap semiconductor devices such as GaN‐ or SiC‐based high‐power electronics. Direct measurements of interfacial thermal resistance and device‐level thermal management performance will be important in future work. Furthermore, the type‐P G/D heterojunction and its unique electrical properties prepared via this approach open a new avenue for the development of diamond‐based semiconductor devices. Our findings not only provide an innovative synthesis method for high‐quality graphene/diamond heterojunctions but also represent a significant step toward integrating diamond materials into the advancement of next‐generation semiconductor technologies.

## 4 Experimental Section/Methods

### 4.1 Fabrication of Graphene/Diamond (G/D) Heterostructures

A home‐built hot‐electron irradiation device was employed, consisting of four components: a water cooling system, a process gas pipeline system, a coating chamber, and a control system. The coating chamber of this system has a diameter of 80 cm and a height of 80 cm, with an effective temperature field area of 24 cm × 20 cm, an ultimate vacuum of 1.0 × 10^−2^ Pa, and a constant‐temperature circulating cooling water system integrated into the chamber outer surface and sample stage.

The hot‐electron irradiation process used in this work is distinct from conventional electron‐beam irradiation methods. In a transmission electron microscope or standard electron‐beam treatment system, electrons are typically accelerated to keV‐level energies and focused into a microscopic beam, leading to highly localized energy deposition, knock‐on damage, radiolysis, or local structural modification [37]. In contrast, the present system uses hot electrons thermionically emitted from molybdenum filaments under high‐temperature operation [57, 58]. These electrons are not focused into a microscopic beam but are distributed over the effective processing area and guided toward the diamond substrate by a positive substrate bias. The process is conducted under a low‐pressure hydrogen atmosphere and coupled with radiative heating from the hot filaments. As a result, the sample experiences the combined effects of moderate electron bombardment, thermal activation, and gas‐surface interaction, rather than a purely high‐energy electron‐beam effect.

The substrates used were high‐pressure high‐temperature (HPHT)‐synthesized (111)‐oriented single‐crystal diamond with a misorientation angle of 3° (dimensions: 3 × 3 mm^2^, thickness: 0.3 mm). Prior to fabrication, the (111) diamond substrates were ultrasonically cleaned in anhydrous ethanol for 10 min, followed by deionized water for 5 min, and then dried with high‐purity nitrogen gas. To eliminate interference from molybdenum filament carburization, the filament surface was pre‐carburized by introducing 50 sccm CH_4_ and 500 sccm H_2_ at a power of 4.6–5 kW for 30 min before loading the substrate. After carburization, the diamond sample was placed in the reactor. To purge residual gases in the deposition and gas‐mixing chambers, 1000 sccm H_2_ was introduced for 10 min prior to the experiment.

The samples were fabricated under the following conditions: hot‐wire power of 6 kW or 7.5 kW, reaction chamber pressure of 8–10 Pa, substrate‐to‐filament distance of 2 cm, positive bias voltage of 200 V applied to the substrate, and heating duration of 30 min. For the fabrication of type‐P heterostructures, the hot‐wire power was set to 6 kW, and the surface temperature (monitored using an infrared thermometer) was controlled within the range of 850–860°C. Under this condition, the relatively mild and spatially extended electron flux enables energy input into the near‐surface and nanoscale subsurface region of diamond. By maintaining the surface temperature below the threshold for extensive cleavage of outermost surface C─C bonds, direct surface graphitization is suppressed, whereas subsurface carbon atoms can be activated. This produces a 2D active layer beneath the surface, which favors the in‐plane transformation of diamond (111) into graphene (0002) and inhibits the competitive formation of type‐V graphitic structures. Upon completion, the hot‐wire power was turned off, the stage was lowered to its initial position, and the sample was allowed to cool naturally under vacuum.

### 4.2 Morphological Characterization

Surface morphologies were characterized using a field‐emission scanning electron microscope (FESEM, Regulus 8100, Hitachi, Japan) operated at an accelerating voltage of 5 kV. Surface height profiles were acquired via atomic force microscopy (AFM, 5000 II, Seiko Instruments, Japan) using a probe tip. Carbon phases were analyzed by Raman spectroscopy (LabRAM Soleil, Horiba, France) utilizing a 532 nm Nd laser with an output power of ∼50 mW. A pristine (111)‐oriented single‐crystal diamond wafer was used as a reference for Raman calibration, exhibiting a characteristic peak at 1332 cm^−^^1^.

### 4.3 Microstructural Characterization

Interfacial microstructures of the G/D composite were examined using transmission electron microscopy (TEM) operated at 200 kV. Additionally, to investigate chemical bonding characteristics, electron energy loss spectroscopy (EELS) was performed on the composite interface. EELS data were acquired using an aberration‐corrected scanning transmission electron microscope (Cs‐corrected STEM, Hitachi HF5000) equipped with a Gatan Quantum 965 EELS system, covering an energy loss range of 235–746 eV. The samples were scanned with a spatial step size of 0.79 nm, energy dispersion of 0.25 eV, exposure time of 0.25 s, and collection semi‐angle of 26.5 mrad.

### 4.4 Piezoresistive Properties

Piezoresistive properties of the G/D heterojunctions were characterized using a custom‐built probe station and a digital source meter (CHI 2450). Figure S9 depicts the measurement configuration: four indium (In) electrodes were prepared at the corners of the graphene surface, and a circular cavity (3 mm in diameter) was integrated beneath the sample stage to enable strain induction under loading. The diamond side faces upward to receive pressure, while the graphene side faces downward and is connected to electrode probes. Herein, electrodes 1–3 and 2–4, aligned along diagonal axes, were selected for analysis. Stepwise loading tests were carried out within the load range of 0–28 N with an interval of 2 N. The applied pressure was calculated from the applied force and the effective indenter contact area of 0.25 mm^2^. Accordingly, the maximum applied pressure was 112 MPa, corresponding to a measurement range of 0–112 MPa. Because the measured GF is sensitive to the loading configuration, device geometry, electrode arrangement, and strain‐evaluation method, the strain used in this work was determined based on the actual loading geometry of the bulk diamond chip [42]. The G/D heterojunction was supported by a 0.3‐mm‐thick single‐crystal diamond substrate, and pressure was applied from the diamond side. Therefore, the deformation was dominated by the elastic response of the diamond substrate, and the strain distribution in the active region could be evaluated by finite element analysis. The GF was then calculated from the relative resistance change measured along the diagonal electrode pairs and the simulated strain. This configuration differs from many flexible graphene‐based films, foams, or polymer‐composite sensors, in which the apparent resistance variation can be affected not only by intrinsic piezoresistivity but also by bending, wrinkling, crack opening/closure, interfacial sliding, contact‐area variation, and reconstruction of percolation pathways [52, 53, 54]. In such systems, the effective strain acting on the conductive network is often difficult to determine precisely, and the reported apparent GF may include structural amplification effects. The present measurement configuration therefore provides a relatively rigid and physically defined platform for evaluating the piezoresistive response of the covalently bonded G/D heterojunction under small elastic deformation.

### 4.5 Density Functional Theory (DFT) Calculations

All calculations were performed within the framework of spin‐polarized density functional theory using the projector augmented plane‐wave (PAW) method, as implemented in the Vienna ab initio simulation package (VASP) [59]. The generalized gradient approximation (GGA) with the Perdew‐Burke‐Ernzerhof (PBE) functional was adopted for the exchange‐correlation potential [60]. Long‐range van der Waals interactions were described using the DFT‐D3 approach [61]. The plane‐wave cutoff energy was set to 480 eV. The convergence criterion for the Kohn‐Sham equation iteration was 10^−5^ eV, and all structures were relaxed until the residual forces on atoms were less than 0.02 eV/Å. Data analysis and visualization were performed using the VASPKIT code [62] and VESTA software [63]. A vacuum spacing of 20 Å was introduced perpendicular to the slab to avoid interlayer interactions.

## Funding

The study is supported by the Specialized Research Fund for State Key Laboratories (Grant No. HYZZ2026T01), the Young Taishan Scholar Program of Shandong Province (No. tsqnz20221149), the Natural Science Foundation of Shandong Province, China (Grant No. ZR2022MF300), Talent Research Project of Qilu University of Technology (Shandong Academy of Sciences) (2024RCKY031). Key R&D Program of Shandong Province, China (2023ZLYS01).

## Conflicts of Interest

The authors declare no conflict of interest.

## Supporting information




**Supporting File**: adma72902‐sup‐0001‐SuppMat.docx.

## Data Availability

The data that support the findings of this study are available from the corresponding author upon reasonable request.

## References

[1] X. Zhang , Y. Zhang , H. Yu , et al., “van der Waals‐Interface‐Dominated All‐2D Electronics,” Advanced Materials 35, no. 50 (2023): 2207966. 10.1002/adma.202207966 36353883

[2] Y. Xiong , D. Xu , Y. Feng , G. Zhang , P. Lin , and X. Chen , “P‐Type 2D Semiconductors for Future Electronics,” Advanced Materials 35, no. 50 (2023): 2206939, 10.1002/adma.202206939. 36245325

[3] X. Guan , Z. Lei , R. Xue , et al., “Polarization: A Universal Driving Force for Energy, Environment, and Electronics,” Advanced Materials 37, no. 1 (2025): 2413525, 10.1002/adma.202413525. 39551991

[4] A. A. Wood , D. J. McCloskey , N. Dontschuk , et al., “3D‐Mapping and Manipulation of Photocurrent in an Optoelectronic Diamond Device,” Advanced Materials 36, no. 40 (2024): 2405338, 10.1002/adma.202405338. 39177116

[5] W. Zhao , G. Chen , T. Teraji , Y. Koide , M. Toda , and M. Liao , “Ultra‐High Sensitivity, Wide‐Range Thermometry Based on High‐Quality Microscale Diamond Resonators,” Advanced Materials 37, no. 42 (2025): 02012, 10.1002/adma.202502012. PMC12548502 40717681

[6] J. Liang , A. Kobayashi , Y. Shimizu , et al., “Fabrication of GaN/Diamond Heterointerface and Interfacial Chemical Bonding State for Highly Efficient Device Design,” Advanced Materials 33, no. 43 (2021): 2104564, 10.1002/adma.202104564. 34498296

[7] E. H. Lock , J. Lee , D. S. Choi , R. G. Bedford , S. P. Karna , and A. K. Roy , “Materials Innovations for Quantum Technology Acceleration: A Perspective,” Advanced Materials 35, no. 27 (2023): 2201064. 10.1002/adma.202201064 37021584

[8] P. Strobel , M. Riedel , J. Ristein , and L. Ley , “Surface Transfer Doping of Diamond,” Nature 430, no. 6998 (2004): 439–441, 10.1038/nature02751. 15269764

[9] A. Denisenko , A. Aleksov , A. Pribil , P. Gluche , W. Ebert , and E. Kohn , “Hypothesis on the Conductivity Mechanism in Hydrogen Terminated Diamond Films,” Diamond and Related Materials 9, no. 3–6 (2000): 1138–1142, 10.1016/S0925-9635(99)00317-9.

[10] W. Fei , T. Bi , M. Iwataki , S. Imanishi , and H. Kawarada , “Oxidized Si Terminated Diamond and Its MOSFET Operation with SiO_2_ Gate Insulator,” Applied Physics Letters 116, no. 21, 212103(2020), 10.1063/1.5143982.

[11] C. L. Patterson , O. I. Sheekey , T. B. Arp , et al., “Superconductivity and Spin Canting in Spin–Orbit‐Coupled Trilayer Graphene,” Nature 641 (2025): 632–638, 10.1038/s41586-025-08863-w. 40335691

[12] Y. Zhang , G. Shavit , H. Ma , et al., “Twist‐Programmable Superconductivity in Spin–Orbit‐coupled Bilayer Graphene,” Nature 641, no. 8063 (2025): 625–631, 10.1038/s41586-025-08959-3. 40335702

[13] Y. Qi , B. Deng , X. Guo , et al., “Switching Vertical to Horizontal Graphene Growth Using Faraday Cage‐Assisted PECVD Approach for High‐Performance Transparent Heating Device,” Advanced Materials 30, no. 8 (2018): 1704839, 10.1002/adma.201704839. 29318672

[14] V. Kuznetsov , I. Zilberberg , Y. V. Butenko , A. Chuvilin , and B. Segall , “Theoretical Study of the Formation of Closed Curved Graphite‐Like Structures During Annealing of Diamond Surface,” Journal of Applied Physics 86, no. 2 (1999): 863–870, 10.1063/1.370816.

[15] F. Pan , K. Ni , Y. Ma , et al., “Phase‐Changing in Graphite Assisted by Interface Charge Injection,” Nano Letters 21, no. 13 (2021): 5648–5654, 10.1021/acs.nanolett.1c01225. 34165978

[16] K. Hembram , S. Lee , H. Im , H. Ju , S.‐H. Jeong , and J.‐K. Lee , “The Surface Hybridization of Diamond with Vertical Graphene: A New Route to Diamond Electronics,” Materials Horizons 7, no. 2 (2020): 470–476, 10.1039/C9MH01588D.

[17] Z. Zhai , C. Zhang , B. Chen , et al., “Covalently‐Bonded Diaphite Nanoplatelet With Engineered Electronic Properties of Diamond,” Advanced Functional Materials 35, no. 21, (2025) 2401949, 10.1002/adfm.202401949.

[18] J.‐K. Lee , S.‐C. Lee , J.‐P. Ahn , S.‐C. Kim , J. I. Wilson , and P. John , “The Growth of AA Graphite on (111) Diamond,” The Journal of Chemical Physics 129, no. 23, (2008): 234709, 10.1063/1.2975333. 19102554

[19] S. Tulic , T. Waitz , M. Caplovicová , et al., “Covalent Diamond–Graphite Bonding: Mechanism of Catalytic Transformation,” ACS Nano 13, no. 4 (2019): 4621–4630, 10.1021/acsnano.9b00692. 30883098 PMC6482437

[20] S. Tulić , T. Waitz , M. Čaplovičová , et al., “Catalytic Graphitization of Single‐Crystal Diamond,” Carbon 185 (2021): 300–313, 10.1016/j.carbon.2021.08.082.

[21] B. Shen , Z. Ji , Q. Lin , et al., “Graphenization of Diamond,” Chemistry of Materials 34, no. 9 (2022): 3941–3947, 10.1021/acs.chemmater.1c04322.

[22] A. V. Okotrub , D. V. Gorodetskiy , Y. N. Palyanov , D. A. Smirnov , and L. G. Bulusheva , “Iron‐Catalyzed Growth of Vertical Graphitic Layers on the (100) Face of Single‐Crystal Diamond,” The Journal of Physical Chemistry C 127, no. 7 (2023): 3563–3569, 10.1021/acs.jpcc.2c08080.

[23] D. Selli , I. Baburin , S. Leoni , Z. Zhu , D. Tománek , and G. Seifert , “Theoretical Investigation of the Electronic Structure and Quantum Transport in the Graphene–C (111) Diamond Surface System,” Journal of Physics: Condensed Matter 25, no. 43 (2013): 435302, 10.1088/0953-8984/25/43/435302. 24096938

[24] W. Hu , Z. Li , and J. Yang , “Diamond as an Inert Substrate of Graphene,” The Journal of Chemical Physics 138, no. 5, 054701 (2013), 10.1063/1.4789420. 23406135

[25] Y. Ma , Y. Dai , M. Guo , and B. Huang , “Graphene‐diamond Interface: Gap Opening and Electronic Spin Injection,” Physical Review B—Condensed Matter and Materials Physics 85, no. 23 (2012): 235448, 10.1103/PhysRevB.85.235448.

[26] B. P. Reed , M. E. Bathen , J. W. R. Ash , et al., “Diamond (111) Surface Reconstruction and Epitaxial Graphene Interface,” Physical Review B 105, no. 20 (2022): 205304, 10.1103/PhysRevB.105.205304.

[27] Y. Song , N. Yang , C. Nebel , and K. Larsson , “Formation Conditions for Epitaxial Graphene on Diamond (111) Surfaces,” Advances in Nanoscience and Nanotechnology (ANN) 2, no. 1, (2018), 00002, 10.33140/ANN/02/01/00002.

[28] N. Tokuda , M. Fukui , T. Makino , D. Takeuchi , S. Yamsaki , and T. Inokuma , “Formation of Graphene‐on‐Diamond Structure by Graphitization of Atomically Flat Diamond (111) Surface,” Japanese Journal of Applied Physics 52, no. 11R (2013): 110121, 10.7567/JJAP.52.110121.

[29] A. De Vita , G. Galli , A. Canning , and R. Car , “A Microscopic Model for Surface‐Induced Diamond‐to‐Graphite Transitions,” Nature 379, no. 6565 (1996): 523–526, 10.1038/379523a0.

[30] L. Sun , M. A. Marques , and S. Botti , “Prediction and Characterization of Graphitic Structures at Diamond Grain Boundaries,” The Journal of Physical Chemistry C 126, no. 35 (2022): 15019–15029, 10.1021/acs.jpcc.2c03788.

[31] K. Luo , B. Liu , W. Hu , et al., “Coherent Interfaces Govern Direct Transformation From Graphite to Diamond,” Nature 607, no. 7919 (2022): 486–491, 10.1038/s41586-022-04863-2. 35794481 PMC9300464

[32] S. Fan , S. Xiao , H. Zhang , et al., “High‐Temperature Friction and Oxidation Resistance of Self‐Sacrificial Diamond‐Graphene Heterostructures Coatings,” Carbon 235 (2025): 120072, 10.1016/j.carbon.2025.120072.

[33] P. Németh , K. McColl , R. L. Smith , et al., “Diamond‐Graphene Composite Nanostructures,” Nano Letters 20, no. 5 (2020): 3611–3619, 10.1021/acs.nanolett.0c00556. 32267704 PMC7227005

[34] T. Apostolova , V. Kurylo , and I. Gnilitskyi , “Ultrafast Laser Processing of Diamond Materials: A Review,” Frontiers in Physics 9 (2021): 650280, 10.3389/fphy.2021.650280.

[35] C. Wang , K. Ho , M. Shirk , and P. Molian , “Laser‐induced Graphitization on a Diamond (111) Surface,” Physical Review Letters 85, no. 19 (2000): 4092–4095, 10.1103/PhysRevLett.85.4092. 11056632

[36] B. Ali , H. Xu , D. Chetty , R. T. Sang , I. V. Litvinyuk , and M. Rybachuk , “Laser‐induced Graphitization of Diamond Under 30 Fs Laser Pulse Irradiation,” The Journal of Physical Chemistry Letters 13, no. 12 (2022): 2679–2685, 10.1021/acs.jpclett.2c00429. 35302380

[37] R. Egerton , “Control of Radiation Damage in the TEM,” Ultramicroscopy 127 (2013): 100–108, 10.1016/j.ultramic.2012.07.006. 22910614

[38] A. C. Ferrari and D. M. Basko , “Raman Spectroscopy as a Versatile Tool for Studying the Properties of Graphene,” Nature Nanotechnology 8, no. 4 (2013): 235–246, 10.1038/nnano.2013.46. 23552117

[39] D. Chen , G. Chen , L. Lv , et al., “General Approach for Synthesizing Hexagonal Diamond by Heating Post‐graphite Phases,” Nature Materials 24, no. 4 (2025): 513–518, 10.1038/s41563-025-02126-9. 39929964

[40] D. A. Muller , Y. Tzou , R. Raj , and J. Silcox , “Mapping sp2 and sp3 States of Carbon at Sub‐Nanometre Spatial Resolution,” Nature 366, no. 6457 (1993): 725–727, 10.1038/366725a0.

[41] L. A. Garvie , P. Németh , and P. R. Buseck , “Transformation of Graphite to Diamond via a Topotactic Mechanism,” American Mineralogist 99, no. 2–3 (2014): 531–538, 10.2138/am.2014.4658.

[42] A. A. Barlian , W.‐T. Park , J. R. Mallon , A. J. Rastegar , and B. L. Pruitt , “Review: Semiconductor Piezoresistance for Microsystems,” Proceedings of the IEEE 97, no. 3 (2009): 513–552, 10.1109/JPROC.2009.2013612. 20198118 PMC2829857

[43] X. Han , M. Huang , Z. Wu , et al., “Advances in High‐Performance MEMS Pressure Sensors: Design, Fabrication, and Packaging,” Microsystems & Nanoengineering 9, no. 1 (2023): 156, 10.1038/s41378-023-00620-1. 38125202 PMC10730882

[44] T. Nguyen , T. Dinh , A. R. M. Foisal , et al., “Giant Piezoresistive Effect by Optoelectronic Coupling in a Heterojunction,” Nature Communications 10, no. 1, (2019): 4139, 10.1038/s41467-019-11965-5. PMC6742666 31515479

[45] H.‐P. Phan , D. Viet Dao , P. Tanner , et al., “Fundamental Piezoresistive Coefficients of p‐type Single Crystalline 3C‐SiC,” Applied Physics Letters 104, no. 11, (2014): 111905, 10.1063/1.4869151.

[46] S. Sahli and D. Aslam , “Ultra‐high Sensitivity Intra‐grain Poly‐Diamond Piezoresistors,” Sensors and Actuators A: Physical 71, no. 3 (1998): 193–197, 10.1016/S0924-4247(98)00181-2.

[47] M. Deng , X. Zhang , K. Fang , Z. Gai , Y. Zhou , and Y. Yang , “Correlation of Residual Stress on Piezoresistive Properties of Boron‐Doped Diamond Films,” Diamond and Related Materials 150 (2024): 111677, 10.1016/j.diamond.2024.111677.

[48] J. Zhou , Y. Gu , P. Fei , et al., “Flexible Piezotronic Strain Sensor,” Nano Letters 8, no. 9 (2008): 3035–3040, 10.1021/nl802367t. 18707178

[49] S. Manzeli , A. Allain , A. Ghadimi , and A. Kis , “Piezoresistivity and Strain‐Induced Band Gap Tuning in Atomically Thin MoS_2_ ,” Nano Letters 15, no. 8 (2015): 5330–5335, 10.1021/acs.nanolett.5b01689. 26191965

[50] R. J. Grow , Q. Wang , J. Cao , D. Wang , and H. Dai , “Piezoresistance of Carbon Nanotubes on Deformable Thin‐Film Membranes,” Applied Physics Letters 86, no. 9 (2005): 093104, 10.1063/1.1872221.

[51] N.‐K. Chang , C.‐C. Su , and S.‐H. Chang , “Fabrication of Single‐Walled Carbon Nanotube Flexible Strain Sensors with High Sensitivity,” Applied Physics Letters 92, no. 6, (2008): 063501, 10.1063/1.2841669.

[52] M. Cao , J. Su , S. Fan , H. Qiu , D. Su , and L. Li , “Wearable Piezoresistive Pressure Sensors Based on 3D Graphene,” Chemical Engineering Journal 406 (2021): 126777, 10.1016/j.cej.2020.126777.

[53] A. D. Smith , F. Niklaus , A. Paussa , et al., “Piezoresistive Properties of Suspended Graphene Membranes Under Uniaxial and Biaxial Strain in Nanoelectromechanical Pressure Sensors,” ACS Nano 10, no. 11 (2016): 9879–9886, 10.1021/acsnano.6b02533. 27797484 PMC5138005

[54] S. Zheng , X. Wu , Y. Huang , et al., “Multifunctional and Highly Sensitive Piezoresistive Sensing Textile Based on a Hierarchical Architecture,” Composites Science and Technology 197 (2020): 108255, 10.1016/j.compscitech.2020.108255.

[55] A. del Bosque , X. F. Sánchez‐Romate , A. Gómez , M. Sánchez , and A. Urena , “Highly Stretchable Strain Sensors Based on Graphene Nanoplatelet‐Doped Ecoflex for Biomedical Purposes,” Sensors and Actuators A: Physical 353 (2023): 114249.

[56] A. del Bosque , X. F. Sánchez‐Romate , M. Sánchez , and A. Urena , “Ultrasensitive and Highly Stretchable Sensors for Human Motion Monitoring Made of Graphene Reinforced Polydimethylsiloxane: Electromechanical and Complex Impedance Sensing Performance,” Carbon 192 (2022): 234–248.

[57] J. E. Butler , Y. A. Mankelevich , A. Cheesman , J. Ma , and M. Ashfold , “Understanding the Chemical Vapor Deposition of Diamond: Recent Progress,” Journal of Physics: Condensed Matter 21, no. 36 (2009): 364201. 21832307 10.1088/0953-8984/21/36/364201

[58] Q. Chen and Z. Lin , “Electron‐Emission‐Enhanced Diamond Nucleation on Si by Hot Filament Chemical Vapor Deposition,” Applied Physics Letters 68, no. 17 (1996): 2450–2452.

[59] G. Kresse and J. Furthmüller , “Efficient Iterative Schemes for Ab Initio Total‐Energy Calculations Using a Plane‐Wave Basis Set,” Physical Review B 54, no. 16 (1996): 11169–11186, 10.1103/PhysRevB.54.11169. 9984901

[60] J. P. Perdew , K. Burke , and M. Ernzerhof , “Generalized Gradient Approximation Made Simple,” Physical Review Letters 77, no. 18 (1996): 3865–3868, 10.1103/PhysRevLett.77.3865. 10062328

[61] S. Grimme , J. Antony , S. Ehrlich , and H. Krieg , “A Consistent and Accurate Ab Initio Parametrization of Density Functional Dispersion Correction (DFT‐D) for the 94 Elements H‐Pu,” The Journal of Chemical Physics 132, no. 15, (2010): 154104, 10.1063/1.3382344. 20423165

[62] V. Wang , N. Xu , J.‐C. Liu , G. Tang , and W.‐T. Geng , “VASPKIT: A User‐friendly Interface Facilitating High‐throughput Computing and Analysis Using VASP Code,” Computer Physics Communications 267 (2021): 108033, 10.1016/j.cpc.2021.108033.

[63] K. Momma and F. Izumi , “VESTA: A Three‐Dimensional Visualization System for Electronic and Structural Analysis,” Journal of Applied Crystallography 41, no. 3 (2008): 653–658, 10.1107/S0021889808012016.

