# Supplementary material for: Graphene (0002)/Diamond (111) Heterojunction with High Piezoresistive Response
Source: Adv Sci (Weinh). 2026 Jul 6:e76096. Online ahead of print. doi: 10.1002/advs.76096 (PMC13335436; doi:10.1002/advs.76096)
Supplement: Supplementary file 1 — Supporting File: adma72902‐sup‐0001‐SuppMat.docx. [file ADVS-9999-e76096-s001.docx]

Supporting Information

Graphene (0002)/Diamond (111) Heterojunction with High Piezoresistive Response

Xueyu Zhang, Kun Guo, Zhigang Gai*, Tianxiao Guo, Yuan Gao, Jiancai Leng, Mei Zhang, Tonggang Jiu, Yibao Wang, Shousheng Liu, Xin Jiang*


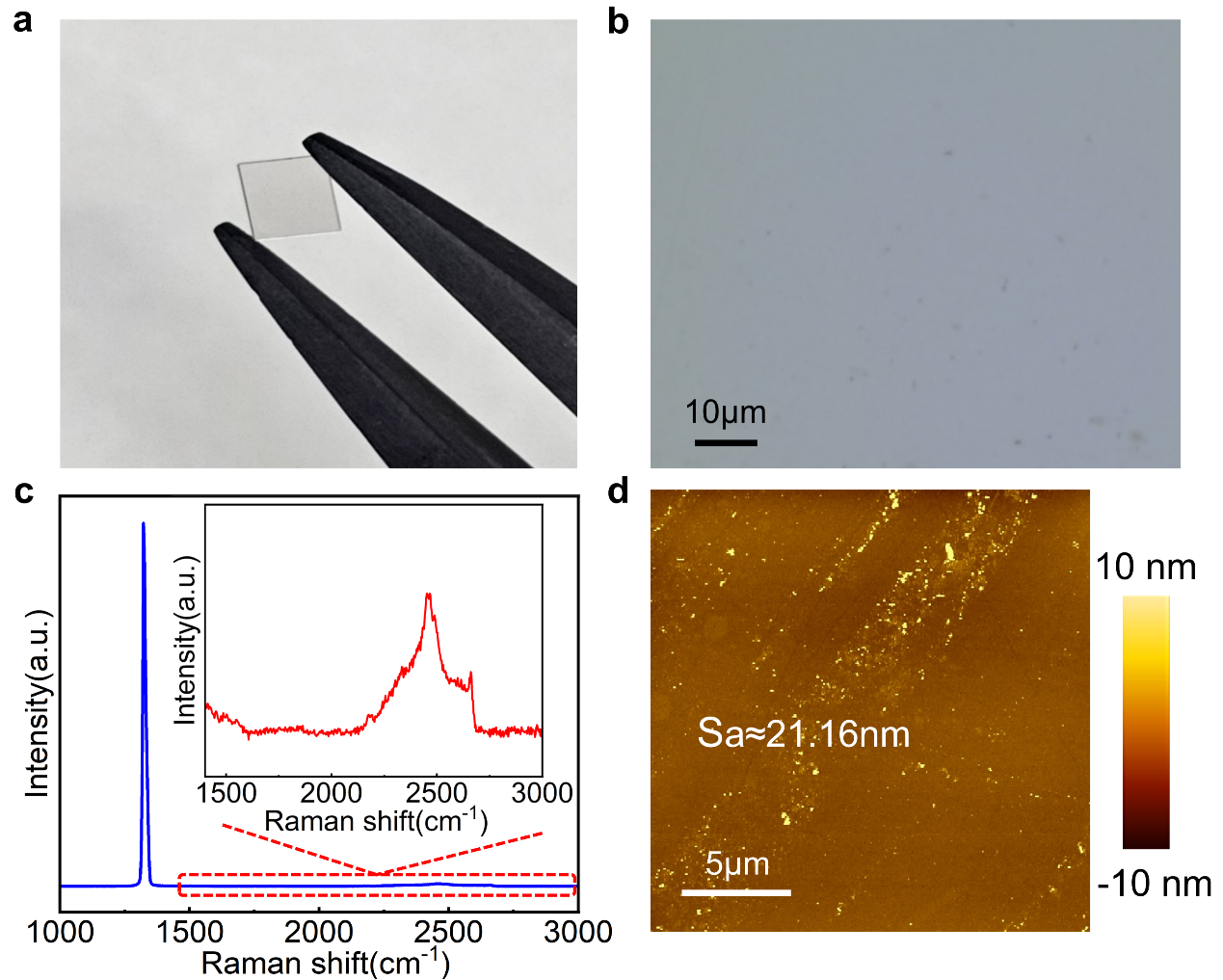


**Figure S1. Single-crystal diamond surface morphology. a)** Photograph of intrinsic single-crystal diamond (3×3×0.3 mm), **b**) Optical microscopy image of intrinsic single-crystal diamond, **c**) Raman spectrum of single-crystal diamond, **d**) Atomic force microscopy (AFM) image of single-crystal diamond.


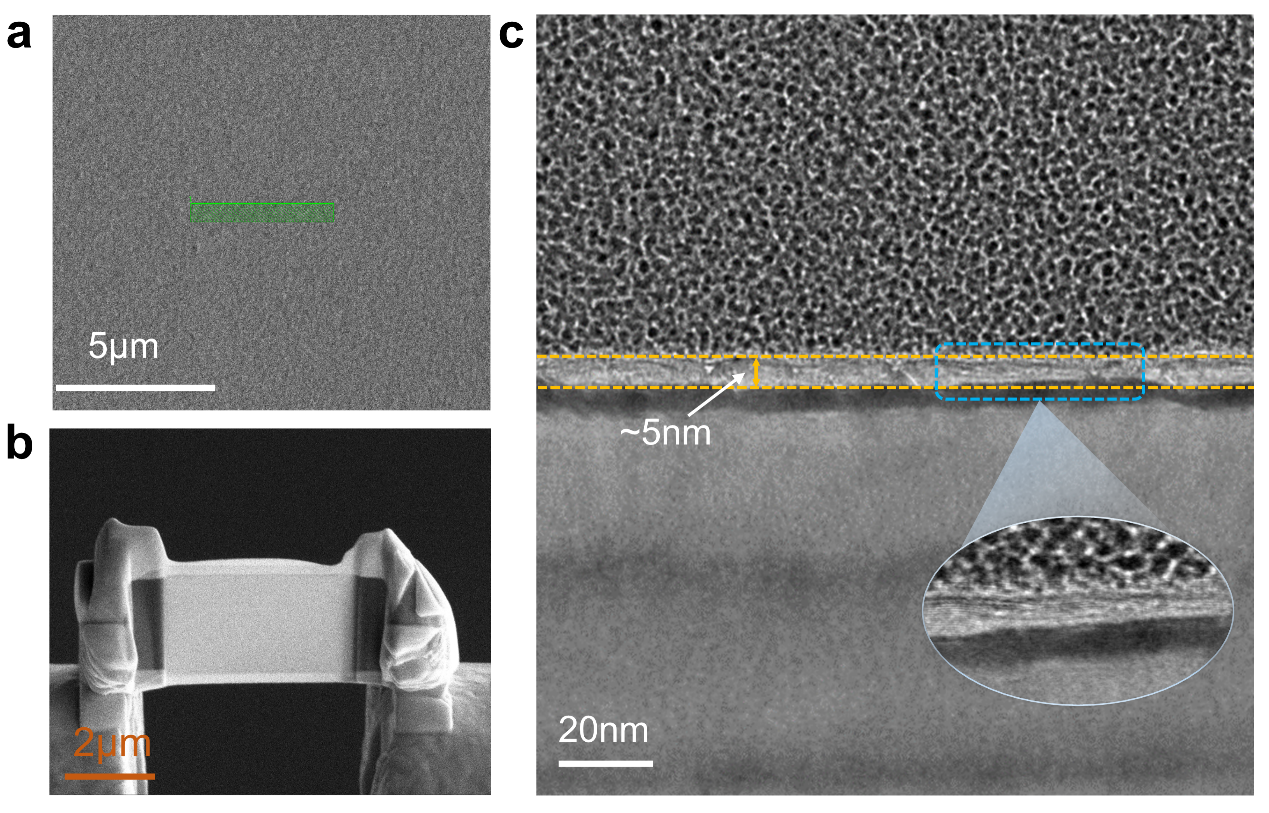


**Figure S2. Preparation of type-P G/D heterojunction TEM samples. a**) FIB selected-area image of the type-P G/D heterojunction surface, **b**) FIB sample preparation photograph, **c**) Cross-sectional TEM image of the type-P G/D structure.


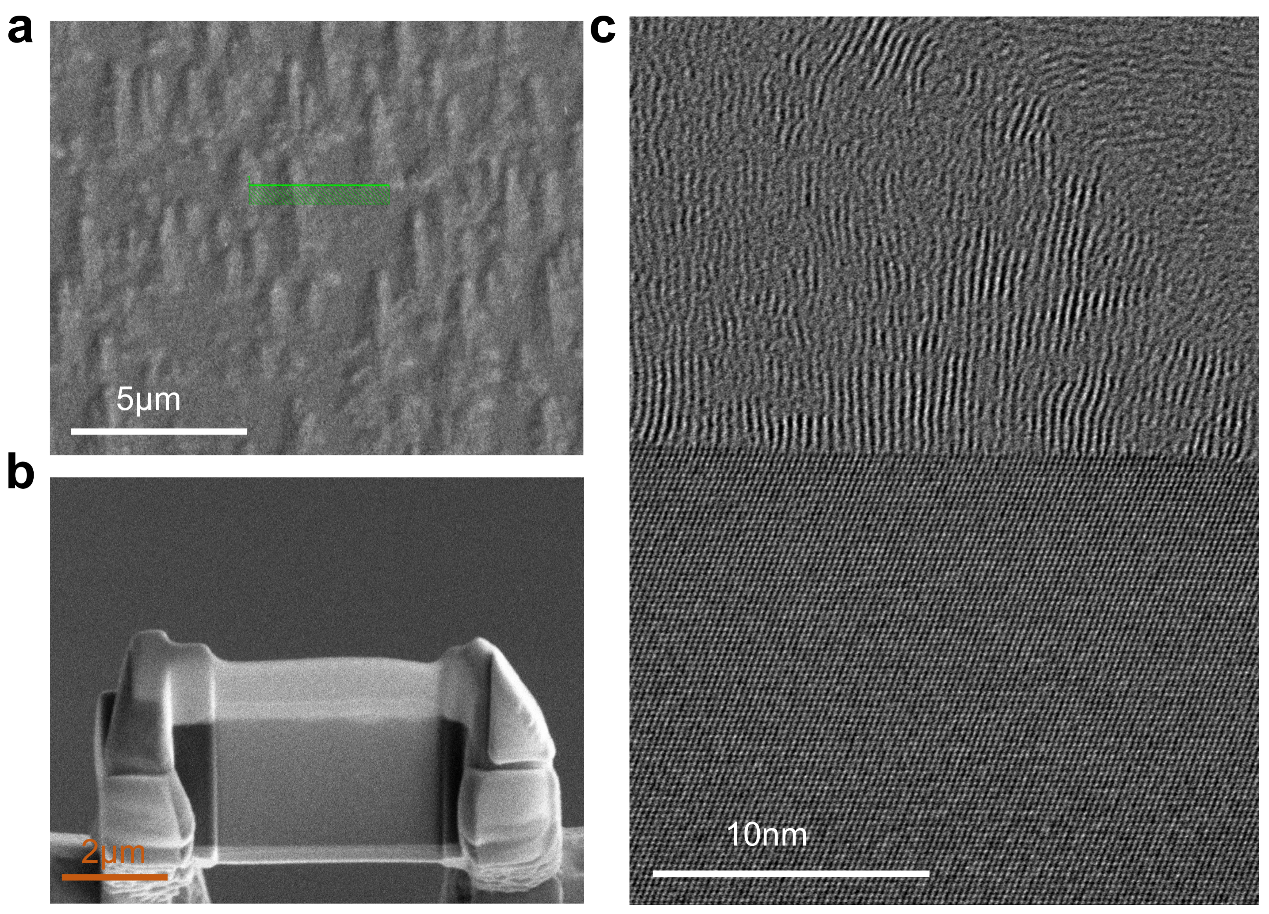


**Figure S3. Preparation of type-V G/D heterojunction TEM samples. a)** FIB selected-area image of the type-V G/D heterojunction surface, **b**) FIB sample preparation photograph, **c**) Cross-sectional TEM image of the type-V G/D heterojunction.


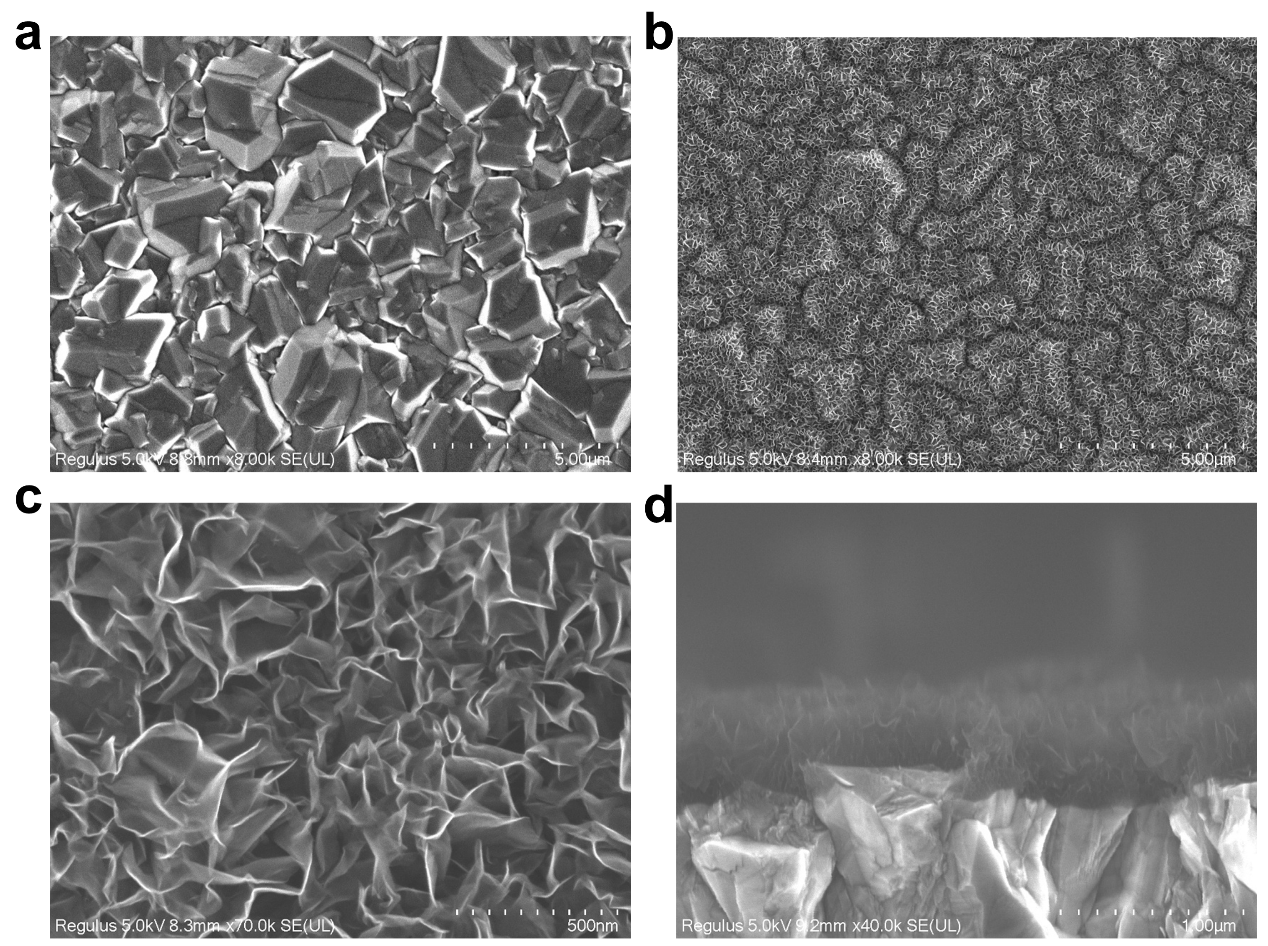


**Figure S4. Preparation of G/D heterojunction by CVD method. a)** SEM Image of Polycrystalline Diamond. **b)** and **c)** SEM Morphologies of the G/D Heterojunction Surface at Different Magnifications. **d)** SEM Morphology of the G/D Heterojunction Cross-Section. In the experiment of preparing G/D heterojunctions on the surface of polycrystalline diamond with externally introduced CH_4_ as the carbon source, different crystal planes on the polycrystalline surface all exhibit a type-V structure.


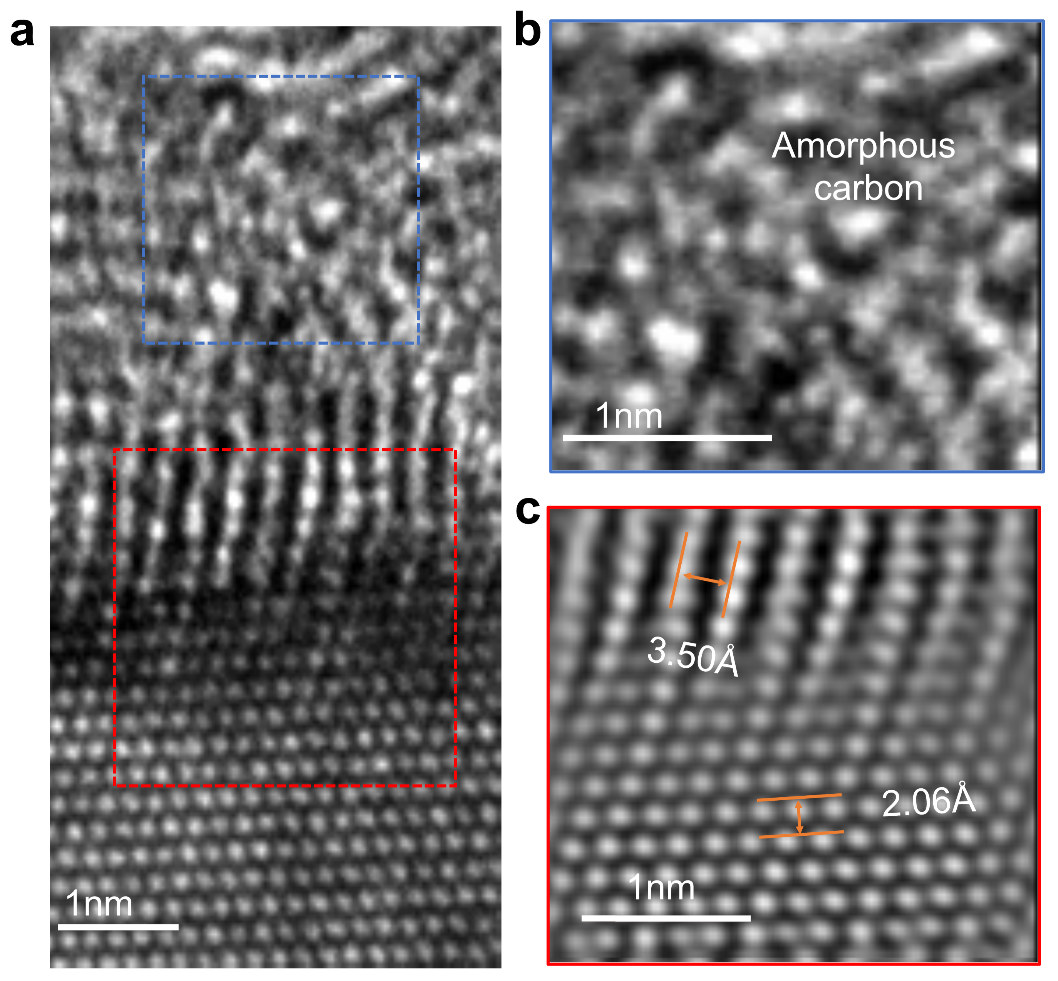


**Figure S5. TEM of the G/D heterojunction fabricated at a surface experimental temperature of 880 ℃**. **a)** Cross-sectional TEM image of the G/D heterojunction structure, **b**) High-resolution image of the blue region, **c**) High-resolution image of the red region.


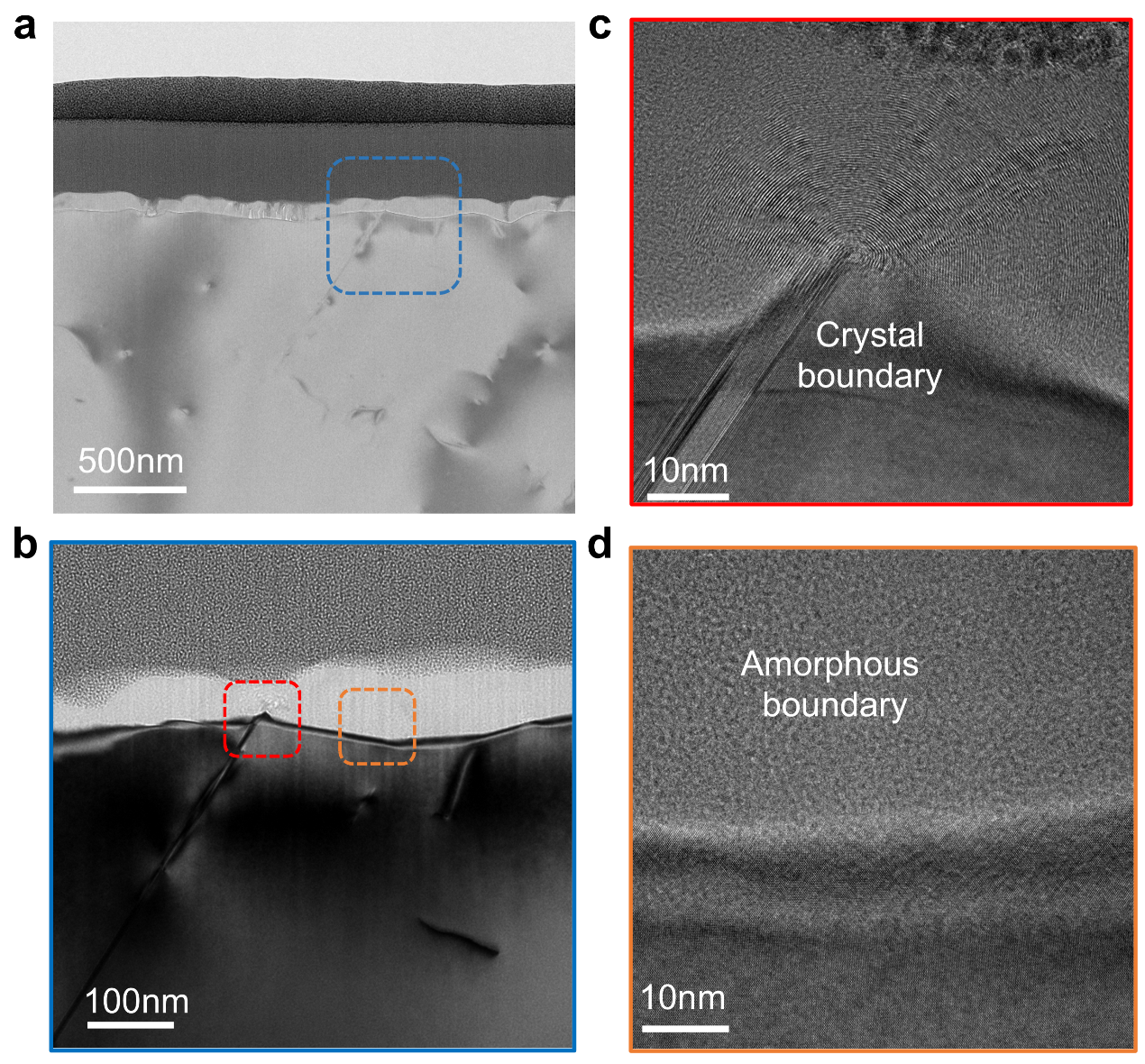


**Figure S6. The influence of surface defects on heterojunction interface structure. a)** and **b)** Cross-sectional TEM images of the G/D heterojunction, **c**) High-resolution image of the interface at the grain boundary, and **d**) High-resolution image of the obtained amorphous carbon structure.


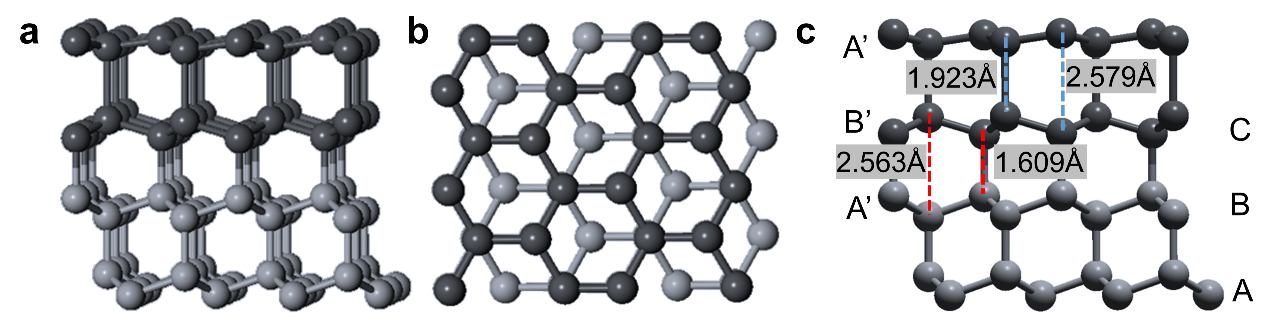


**Figure S7. Theoretical model of** **cubic diamond/hexagonal diamond composite structure. a**) Side view of the cubic diamond/hexagonal diamond composite structure, **b**) Top view of the cubic diamond/hexagonal diamond composite structure, **c**) Crystalline plane stacking sequence of cubic diamond and hexagonal diamond.


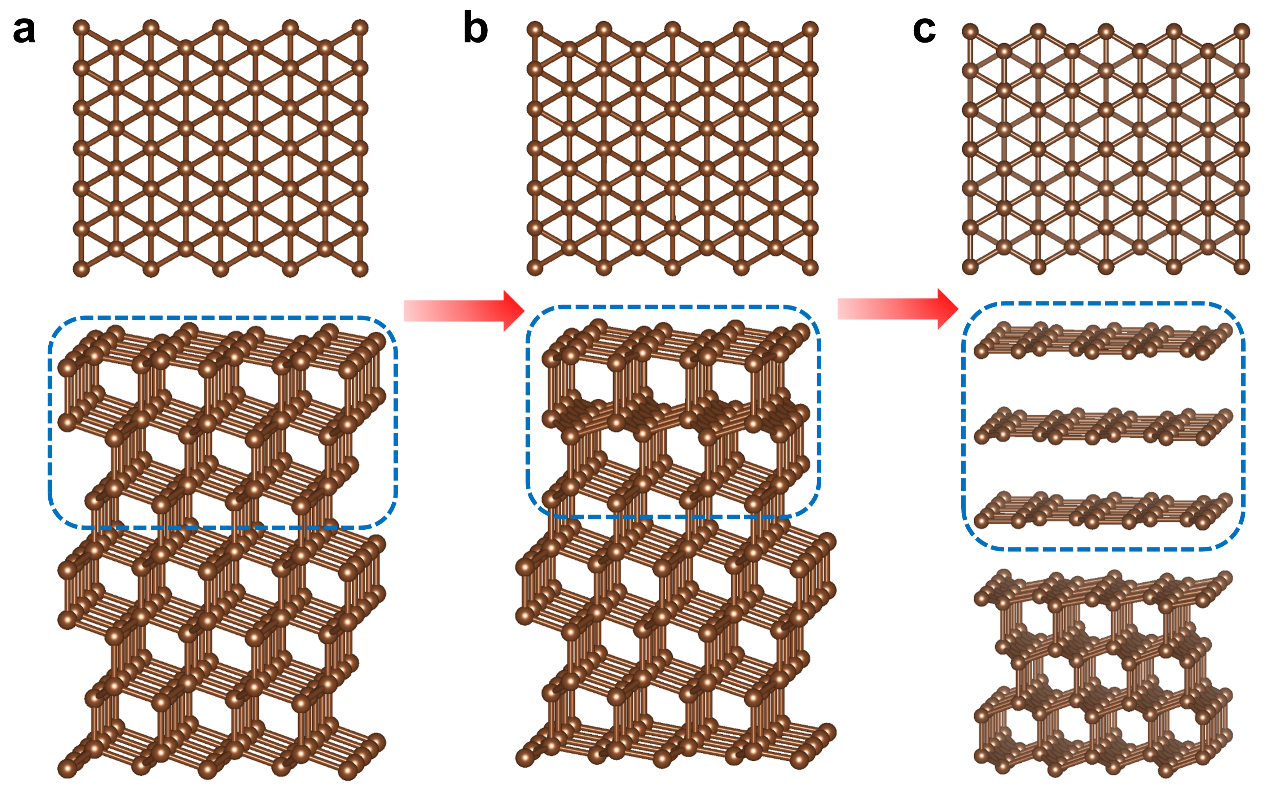


**Figure S8. Theoretical model of the transformation from single-crystal diamond to G/D heterojunction structure. a**) Structural model of cubic diamond, **b**) Structural model of hexagonal diamond (upper three layers) / cubic diamond, **c**) Structural model of graphene / diamond.


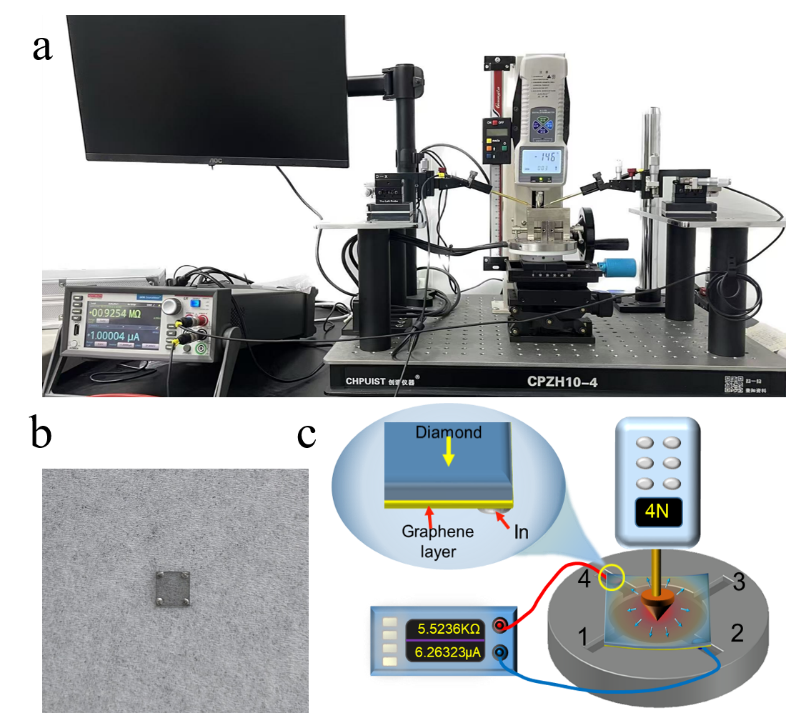


**Figure S9. Piezoresistive property testing equipment. a)** Photograph of the customized equipment for testing the piezoresistive properties of materials. **b)** Photograph of the as-prepared sample. **c)** Schematic of the piezoresistive measurement setup, the diamond side faces upward to receive pressure, while the graphene side faces downward and is connected to electrode probes. The measurement system consists of a high-precision force gauge, a precision displacement stage, a stainless-steel spherical indenter, a Keithley 2400 Source Meter unit, and tungsten carbide probes. The high-precision force gauge is used to record the external load applied to the sample surface in real time, from which the equivalent pressure is calculated using the effective contact area of 0.25 mm². The precision displacement stage controls the loading position of the indenter and the contact position of the probes, ensuring the stability and repeatability of mechanical loading and electrical measurements. The stainless-steel spherical indenter applies mechanical load perpendicularly to the stress-bearing region of the sensing chip. The Keithley 2400 Source Meter unit is used to measure resistance signals, while tungsten carbide probes establish stable electrical contact between the device and the external circuit. After sample fabrication, indium electrodes are deposited at the four corners of the graphene surface to realize reliable electrical connection with the test circuit. All measurements are performed at room temperature (23 °C, relative humidity of 50%). Prior to formal testing, the pressure system is zero-calibrated when the indenter is not in contact with the diamond surface, and the initial resistance of the sample is recorded. Afterwards, stepwise loading tests are carried out within the load range of 0–28 N with an interval of 2 N, corresponding to a pressure range of 0–112 MPa.


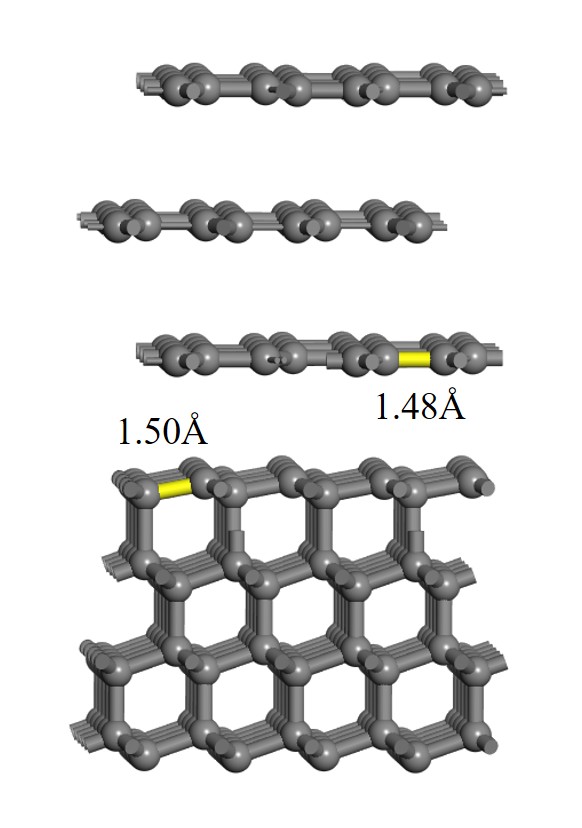


**Figure S10.** **The structural model of the G/D heterojunction obtained by applying a 2% in-plane biaxial tensile strain to** **Figure S8c.**


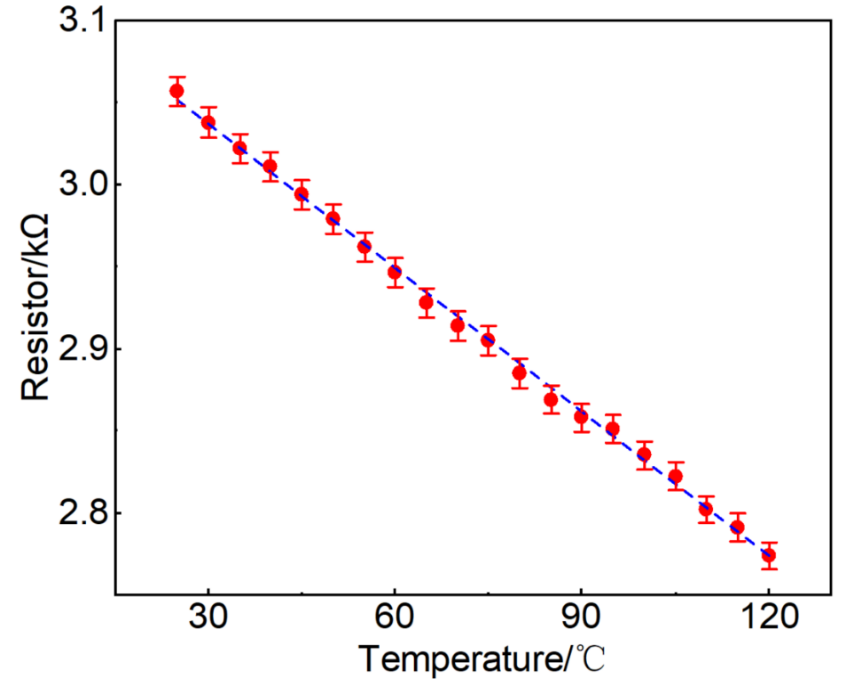


**Figure S11. Temperature‑dependent surface resistance curve of the G/D heterojunction.** The temperature was gradually increased from 25 °C to 120 °C with a step increment of 5 °C. The sample was kept isothermal at each preset temperature point, and its steady-state resistance was recorded after thermal equilibrium was achieved and the resistance stabilized. Repeated tests (10 times) were conducted under identical conditions, and the average values of multiple measurements at each temperature were used for analysis. The results indicate that within the range of 25 °C to 120 °C, the sample resistance decreases nearly linearly with increasing temperature, showing favorable overall linearity. The total resistance change is approximately 284 Ω. This heterojunction exhibits prominent temperature-sensitive response characteristics. A simple calibration algorithm can eliminate the interference of temperature fluctuations on the sensor output signal and effectively suppress measurement errors induced by temperature drift. Accordingly, the pressure sensor fabricated from this heterojunction can maintain stable and reliable performance under various temperature conditions.

**Table S1. Literature survey of piezoresistive performance metrics of leading piezoresistive materials and carbon-based composites.**

| Materials | Testing structure | GF/Sensitivity | Detection limit | Response time |
| --- | --- | --- | --- | --- |
| 3C-SiC/p-Si^[1]^ | cantilever beam | 20/58000 (optoelectronic coupling) | 0.0225-0.0677% | — |
| 3C-SiC^[2]^ | cantilever beam | 30.3 | 0.02 – 0.11 % | — |
| BDD^[3]^ | cantilever beam | 283/4000 (intra-grain) | 0-0.08 % | — |
| BDD^[4]^ | cantilever beam | 257 | 0.003 -0.009 % | — |
| NWs-ZnO^[5]^ | cantilever beam | 1250 (Piezotronic) | 0.2 – 1.0 % | — |
| MoS_2_^[6]^ | suspended / AFM probe | -224 ± 19 | — | — |
| CNT^[7]^ | mechanical pressure | 850 | 0.04 - 0.12 % | — |
| SWCNT^[8]^ | cantilever beam | 269 | 0.004 -0.022 % | — |
| 3DGP^[9]^ | universal testing machine | 448 | 0-50 % | — |
| PDMS/Au^[10]^ | microstructured drain / source electrode | 514 kPa^-1^ | 0-10 Pa | 1.8 ms |
| PANI/rGO/Textile^[11]^ | flexible sensor | -78 | 0.0005-40 kPa | 30 ms |
| PANI/CCS^[12]^ | universal tensile analyzer | 6.72 | 0-95 % | 200 ms |
| GO/SEBS^[13]^ | flexible sensor | 118 | 0-10 % | — |
| Graphene/PDMS^[14]^ | flexible sensor | 15.9 kPa^-1^ | 0−60 kPa | 1.2 ms |
| PGM^[15]^ | flexible sensor | 0.7 kPa^-1^ | 2000 kPa | — |
| Graphene/SiO_2_^[16]^ | suspended-cavity/ vacuum pressure | 6.73 | 0.1-0.5 % | — |
| Graphene^[17]^ | vacuum pressure | 1.14 | 0.1-0.3 % | — |
| UGF/PDMS^[18]^ | tensile strain | 1037 | 0.25-2.0 % | — |
| **Type-P G/D** | **suspended-cavity / mechanical pressure** | **-1149** | **0-41.81MPa** | **33 ms** |

Acronym definitions, boron-doped diamond (BDD), carbon nanotube (CNT), reduced graphene oxide (rGO), styrene−ethylene−butylene−styrene (SEBS), Polyaniline (PANI), polyaniline/cross-linked collagen sponge (PANI/CCS), poly (dimethyl siloxane) (PDMS), three-dimensional graphene-PDMS (3DGP), porous graphene membrane (PGM), ultrathin graphene films (UGF).

References:

[1] T. Nguyen, T. Dinh, A. R. M. Foisal, H.-P. Phan, T.-K. Nguyen, N.-T. Nguyen, D. V. Dao, *Nature communications* **2019**, 10, 4139.

[2] H.-P. Phan, D. Viet Dao, P. Tanner, L. Wang, N.-T. Nguyen, Y. Zhu, S. Dimitrijev, *Applied Physics Letters* **2014**, 104.

[3] S. Sahli, D. Aslam, *Sensors and Actuators A: Physical* **1998**, 71, 193.

[4] M. Deng, X. Zhang, K. Fang, Z. Gai, Y. Zhou, Y. Yang, *Diamond and Related Materials* **2024**, 150, 111677.

[5] J. Zhou, Y. Gu, P. Fei, W. Mai, Y. Gao, R. Yang, G. Bao, Z. L. Wang, *Nano letters* **2008**, 8, 3035.

[6] S. Manzeli, A. Allain, A. Ghadimi, A. Kis, *Nano letters* **2015**, 15, 5330.

[7] R. J. Grow, Q. Wang, J. Cao, D. Wang, H. Dai, *Applied Physics Letters* **2005**, 86.

[8] N.-K. Chang, C.-C. Su, S.-H. Chang, *Applied Physics Letters* **2008**, 92.

[9] M. Cao, J. Su, S. Fan, H. Qiu, D. Su, L. Li, *Chemical Engineering Journal* **2021**, 406, 126777.

[10] Z. Wang, S. Guo, H. Li, B. Wang, Y. Sun, Z. Xu, X. Chen, K. Wu, X. Zhang, F. Xing, *Advanced Materials* **2019**, 31, 1805630.

[11] S. Zheng, X. Wu, Y. Huang, Z. Xu, W. Yang, Z. Liu, M. Yang, *Composites Science and Technology* **2020**, 197, 108255.

[12] L. Liu, Z. Ai, X. Zhang, K. Tang, Y. Pei, *International Journal of Biological Macromolecules* **2024**, 279, 135305.

[13] P. Costa, S. Gonçalves, H. Mora, S. A. Carabineiro, J. C. Viana, S. Lanceros-Mendez, *ACS Applied Materials & Interfaces* **2019**, 11, 46286.

[14] N. Luo, Y. Huang, J. Liu, S. C. Chen, C. P. Wong, N. Zhao, *Advanced materials* **2017**, 29, 1702675.

[15] C. Liu, H. Li, J. Zhao, J. Zhu, X. Huan, Y. Zhang, K. Xu, H. Geng, X. Chen, W. Ding, *Chemical Engineering Journal* **2024**, 494, 153140.

[16] A. D. Smith, F. Niklaus, A. Paussa, S. Schröder, A. C. Fischer, M. Sterner, S. Wagner, S. Vaziri, F. Forsberg, D. Esseni, *ACS nano* **2016**, 10, 9879.

[17] J. Wang, S. Lei, N. Su, W. Hou, M. Li, S. Zheng, H. Zhang, X. Lv, *Applied Physics Letters* **2022**, 121.

[18] X. Li, T. Yang, Y. Yang, J. Zhu, L. Li, F. E. Alam, X. Li, K. Wang, H. Cheng, C. T. Lin, *Advanced Functional Materials* **2016**, 26, 1322.
